# Chain Transfer Kinetics of Rhodixan^®^ A1 RAFT/MADIX Agent

**DOI:** 10.3390/molecules29246004

**Published:** 2024-12-20

**Authors:** Mathias Destarac, Aymeric Guinaudeau, Stéphane Mazières, James Wilson

**Affiliations:** 1 Laboratoire SOFTMAT, CNRS UMR 5623, University of Toulouse, University Toulouse III-Paul Sabatier, 118 Route de Narbonne, 31062 Toulouse Cedex 9, France; aymeric.guinaudeau@syensqo.com (A.G.); stephane.mazieres@univ-tlse3.fr (S.M.); 2 Syensqo, Centre de Recherche et Innovation Lyon, 85 Rue des Frères Perret, BP 62 69192 Saint Fons, France; 3 Syensqo, Centre de Recherche et Innovation Aubervilliers, 52 Rue de la Haie Coq, 93308 Aubervilliers Cedex, France; james.wilson2@syensqo.com

**Keywords:** xanthate, RAFT, radical polymerization, chain transfer, kinetics

## Abstract

Rhodixan^®^ A1 is the trade name for *O*-ethyl *S*-(1-methoxycarbonylethyl)dithiocarbonate, a RAFT/MADIX agent used by Syensqo to produce block copolymer additives for aqueous formulations on an industrial scale. Chain transfer coefficients to Rhodixan^®^ A1 determined for 25 different styrenic, acrylate, and acrylamide monomers were relatively low (0.6 < C_tr_ < 3.8) and evolved in the following order: styrenics < acrylates < acrylamides. The corresponding interchain transfer coefficients followed the same trend (0.6 < C_tr,PnXA1_ < 5.9). In all cases, polymers with predetermined *M_n_* are obtained at high monomer conversion, with dispersities inversely proportional to C_tr,PnXA1_ in most cases. With the added benefit of the established excellent reactivity of Rhodixan^®^ A1 towards unconjugated monomers such as vinyl esters, diallyl, or *N*-vinyl monomers, RAFT/MADIX is a fantastic technological toolbox for tailor-making complex copolymers from monomers of highly disparate reactivities.

## 1. Introduction

Reversible addition−fragmentation chain transfer polymerization (RAFT) recently celebrated its 25th anniversary [1,2]. Significant advances in the knowledge and control of this fascinating technology are frequently observed, and the user community continues to grow [3,4,5,6,7,8]. The establishment of appropriate conditions for the efficient RAFT process of a given monomer relies on the suitable choice of the thiocarbonylthio RAFT chain transfer agent of general structure R-S (C = S)-Z. Some monomer/RAFT agent combinations are known to induce specific (mostly undesired) effects, e.g., polymer retardation or inhibition with dithiobenzoates (Z = Ph) depending on the monomer [9], initialization phenomenon (formation of RAFT agent: monomer 1:1 adduct in the early stages of polymerization) [10] and thermally induced Z-group elimination occur as a side reaction during polymerization [11,12]. In addition, a proper selection of the R leaving group and Z activating group is critical for the fine-tuning of chain transfer kinetics, which dictates the evolution of number-average molar mass (*M_n_*) and dispersity (*Đ*) during polymerization [13,14]. Among the main classes of RAFT agents, xanthates (Z = OR′, coined MADIX agents Samir Zard and Rhodia, MADIX standing for Macromolecular Design by Interchange of Xanthates) [15,16] gained increasing attention over the years and are today firmly established as best suited for the controlled polymerization of unconjugated monomers. In particular, *O*-ethyl xanthates became very popular over time owing to their easy synthesis from commercially available potassium *O*-ethyl xanthate salt and an alkylating agent. Unconjugated monomers whose RAFT/MADIX polymerization is mediated with *O*-ethyl xanthates are numerous, including vinyl esters [17,18,19], *N*-vinyl amides [20,21], *N*-vinylimidazolium salts [22], vinyl phosphonates [23], *S*-vinyl sulfides [24], vinyl sulfonate esters [25], vinyl chloride [26], diallyldimethylammonium chloride [27], and ethylene [28] among others [16,29,30]. In contrast, *O*-ethyl xanthates exhibit moderate reactivity towards conjugated monomers like styrene and derivatives [31,32,33], acrylates [34] and acrylamides [35], and are inefficient for 1,1-disubstituted monomers like methacrylates [36]. In a singular way, the intermediate reactivity of xanthates allows the preparation of block copolymers made of conjugated and non-conjugated monomers [37,38] without resorting to traditional approaches using switchable RAFT agents [39].

**Scheme 1:**
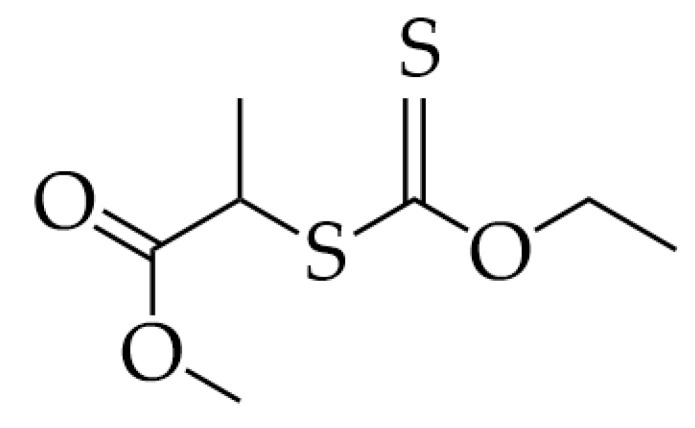
Rhodixan^®^ A1 RAFT/MADIX agent.

*O*-ethyl *S*-(1-methoxycarbonylethyl)dithiocarbonate (XA1, Scheme 1) is one of the few RAFT agents used on an industrial scale. It was registered under the tradename Rhodixan^®^ A1 by Rhodia (now Syensqo), with first commercial success of RAFT/MADIX technology during 2000s for Rhodibloc^®^ RS, an amphiphilic block copolymer with improved stabilizing properties for inverse emulsions, especially under high shear conditions. Rhodibloc^®^ FL fluid loss block copolymer additives were developed during the 2010s for cementing applications for the Oilfield market.

Access to kinetic data for chain transfer events involving RAFT agents can be very valuable, either for modeling RAFT processes (e.g., using Predici software, https://www.cit-wulkow.de/products/predici-polymerization (accessed on 31 October 2024)) [40] or anticipating macromolecular characteristics (*M_n_*, *Đ*) using simpler kinetic analysis of controlled/living polymerizations [41]. Faced with the lack of kinetic data available in the literature, particularly for conjugated monomers, we determined chain transfer coefficients related to Rhodixan^®^ A1 for a series of styrenic, acrylate, and acrylamido monomers of interest.

## 2. Results

### 2.1. Definition of Chain Transfer Coefficients

**Scheme 2:**
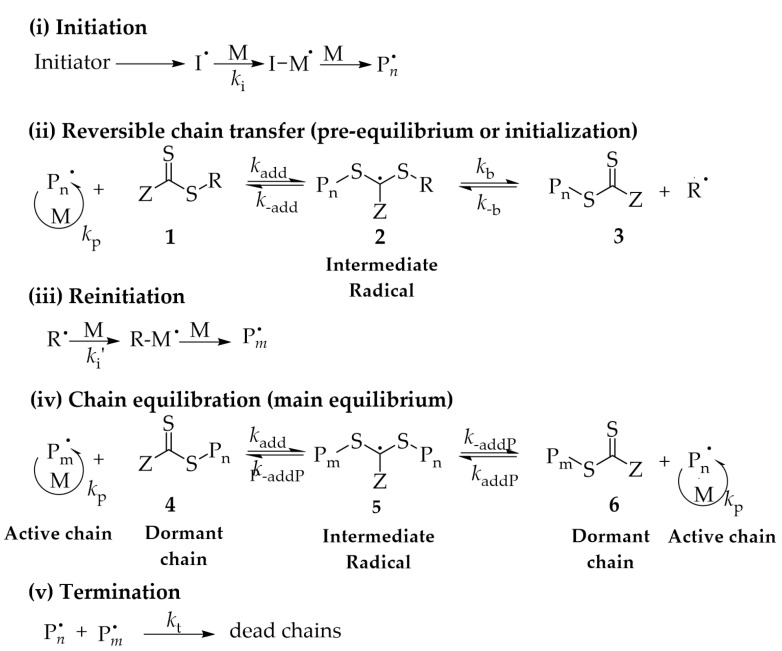
Mechanism of RAFT/MADIX polymerization showing initiation, propagation, reversible chain transfer, reinitiation, chain equilibration and termination steps. Explanatory details can be found in [14].

The reaction steps encountered in a RAFT are recalled in Scheme 2 (illustrated with Z = OEt) for a clear definition of kinetic parameters.

The rate of consumption of the RAFT agent depends on two transfer coefficients: C_tr_ (=*k_tr_*/*k*_p_) and C_−tr_ (=*k*_−tr_/*k*_i_), which describe the reactivity of the propagating radical (P_n_•) and the expelled radical (R•) towards the thiocarbonylthio moieties, respectively. The rate coefficient for chain transfer (*k_tr_*) is defined by the product of the rate coefficient for addition to the RAFT agent (*k*_add_) and a coefficient which represents how the adduct is partitioned between the products and starting materials (Equation (1))

(1) $$k_{tr}=k_{add}\frac{k_{\beta }}{k_{\beta }+k_{-add}}.$$

In a similar fashion, *k*_−tr_ is defined as follows for the reverse process (Equation (2))

(2) $$k_{-tr}=k_{-\beta }\frac{k_{-add}}{k_{\beta }+k_{-add}}.$$

The reverse process cannot be neglected when low k_p_ monomers like styrene (St) or highly reactive RAFT agents (e.g., dithiobenzoates) are used. Moad et al. suggested that when C_−tr_ was ignored and set to zero, the experimental C_tr_ values should be termed apparent coefficients C_tr_^app^ [42]. They proposed that C_−tr_ could be neglected when the independence of C_tr_ on the RAFT agent concentration was established. In the present study, we followed this recommendation considering that C_tr_ values to XA1 for conjugated monomers are low and were shown to be independent on XA1 concentration for a series of acrylates taken as an example (Table S1, see Supplementary Materials). During polymerization, the propagating radicals P_n_• and P_m_• have similar lengths and reactivities. Therefore, the intermediate radical species **5** is symmetrical and k_appP_ and k_−addP_ are supposed to be equal. It appears that the rate coefficient for reversible chain transfer (interchain transfer) k_tr,PnRAFT_ is primarily a function of the rate coefficient for addition to the RAFT terminal group (Equation (3)). The magnitude of interchain transfer depends on the interchain transfer coefficient C_tr,PnRAFT_ (=k_tr,PnRAFT_/k_p_).

(3) $$k_{tr,PnRAFT}=k_{addP}\frac{k_{-addP}}{k_{-addP}+k_{-addP}}=\frac{k_{addP}}{2}$$

### 2.2. Chain Transfer Coefficients Determination Methods

A number of methods have been reported for determining values of the transfer coefficients to RAFT agent. Conventional approaches, such as the Mayo method [43], allow access to C_tr_ by plotting 1/DP_n_ as a function of [RAFT]_0_/[M]_0_. In reality, $C_{tr}{\left(1-x\right)}^{C_{tr}-1}$ (with x = monomer conversion) is obtained and C_tr_ is not, which makes this approach only valid for low conversion and low transfer coefficients, and therefore not appropriate for most of the RAFT agents. An alternative strategy for determining C_tr_ is based on the kinetic model developed by Müller et al. [41,44], which compares the theoretical *M_n_* (*M_n,th_*, assuming full RAFT agent consumption) and actual *M_n_* > *M_n,th_* (when the unreacted RAFT agent is still present) during polymerization. By doing so, *C*_tr_ is obtained on the basis of one single polymerization experiment. Using this approach, however, the polymerization must be monitored usually by size exclusion chromatography (SEC) at conversions where the RAFT agent consumption has not yet been completed, which makes this method not suitable for high C_tr_ values. In addition, the model does not take into account the contribution of initiator-derived chains, which tends to slightly overestimate C_tr_ values. The most used and preferred technique for measuring C_tr_ is based on Equation (4), which is an expression of the evolution of RAFT agent and monomer concentration as a function of C_tr_. The double-log regression of Equation (4) gives direct access to C_tr_ in one single experiment [42]. This method is known to be more accurate than the others because it is solely based on the measurement of the concentrations of RAFT agent and monomer over time, for instance by high performance liquid chromatography.

(4) $$\frac{\left[\mathrm{RAFT}\right]}{{\left[\mathrm{RAFT}\right]}_{0}}={\left(\frac{\left[M\right]}{{\left[M\right]}_{0}}\right)}^{C_{\mathrm{tr}}},$$

In order to determine the interchange transfer coefficient C_tr,PnRAFT_, we applied the SEC-based peak resolution method developed by Fukuda et al. [45].

### 2.3. Styrenic Monomers

**Scheme 3:**
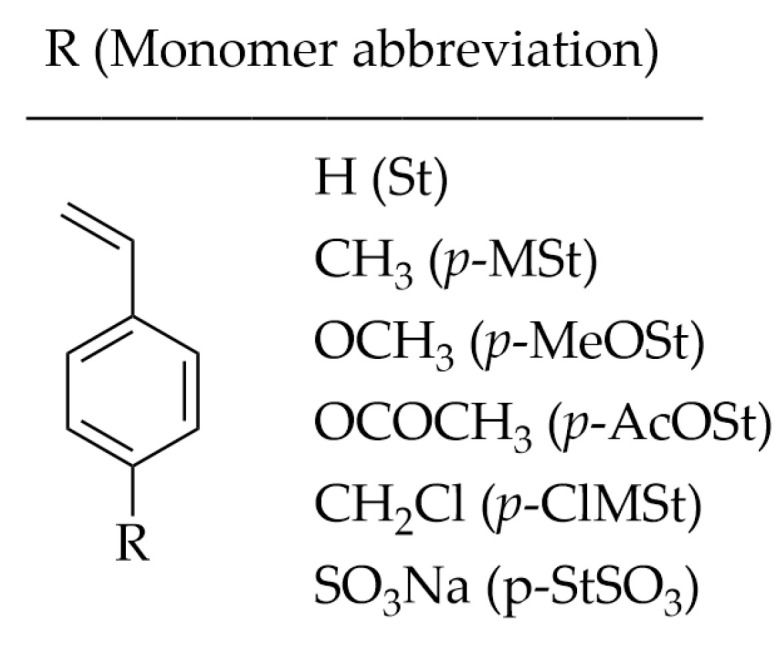
Styrenic monomers of the study.

RAFT/MADIX polymerization of styrene (St) and five *p*-substituted styrenic monomers (Scheme 3) was investigated at 60 °C with XA1. *p*-methylstyrene (*p*-MSt), *p*-methoxystyrene (*p*-MeOSt), *p*-acetoxystyrene (*p*-AcOSt), and *p*-chloromethylstyrene (*p*-ClMSt) were polymerized in bulk whereas sodium 4-styrene sulfonate (*p*-StSO_3_) polymerization was carried out in a 50 wt.% water/ethanol mixture (4.5:1). Substituents in para position of the aromatic ring were selected for their different electron-withdrawing (OCOCH_3_, CH_2_Cl, SO_3_Na) and donating (CH_3_, OCH_3_) character.

The ratio of initial monomer and XA1 concentrations was established for all the hydrophobic monomers of the series so that it corresponds to *M_n,th_*~10,000 g/mol. SEC data for hydrophilic *p*-StSO_3_ are not shown, owing to poly(ethylene oxide) (PEO) calibration which did not give reliable molar masses due to large differences in hydrodynamic volumes between PEO standards and polymer analytes. The evolution of *M_n_* and dispersities during polymerization of styrenic monomers is presented in Figure S1. In all cases, *M_n_* values are higher than those predicted for an efficient RAFT, and tend to get closer to targeted values for the highest conversions. They lie in the same range for a given conversion, whatever the *p*-substituent. At the same time, *Đ* values are high (>1.5 in most cases and in the 1.8–2.0 range at ~80% conversion). These features are characteristic of a slow chain transfer to XA1, with C_tr_ < 1 if we consider Müller’s kinetic model representing systems based on degenerative transfer processes involving slow equilibria [41,44]. Data reported in Table 1 are in agreement with our observations, with C_tr_ values ranging from 0.58 for St to 1.01 for *p*-MeOSt. The corresponding double log plots are available in Figures S2–S7. For St, the measured value is close to those reported elsewhere with the ethyl ester analog of XA1: C_tr_ = 0.68 [32] and 0.89 [31] at 60 °C using the Mayo method, and 0.80 at 70 °C by applying multi-response regression analysis to the experimental data on the RAFT agent and St conversion, number and mass average molar masses, and end-group functionality [33]. In the latter case, d’Hooge and co-workers calculated that for this St/xanthate system characterized by a low C_tr_ value, the dormant macrospecies are always formed via a single exchange, which implies a transfer behavior as in a conventional free radical polymerization.

Coote at al. [46] measured propagation rate coefficients (k_p_) by pulsed laser polymerization for a series of *p*-substituted monomers (R = OCH_3_, CH_3_, F, Cl, Br) in the 20–40 °C range. It was found that neither the Hammett relationship nor the extended Hammett relationship could quantitatively describe the substituent effects on the rate coefficients, though both could provide a reasonable qualitative description of the trends in the data. However, substituents did not have a pronounced effect on k_p_ values. For instance, at 40 °C, k_p_ = 112 L/mol/s for R = CH_3_, 160 L/mol/s for R = H and 197 L/mol/s for R = Cl. Consequently, it is difficult to discuss the effect of *para* substituents of styrene derivatives on *C_tr_* values owing to too small variations of k_p_ and *C_tr_*.

We applied the SEC-based peak resolution method developed by Fukuda et al. [23] to determine the activation rate coefficient k_act_ for a low molar mass PSt-S(C = S)OEt (PSt-XA1) in St polymerization. The data points from Figure S8a,b allowed the calculation of C_tr,PnXA1_ = 0.64 (Figure S8c). This value is very close to C_tr_^app^ (0.58), which can be interpreted by similar leaving group abilities of R in XA1 and PSt chain (Scheme 2). This result is synonymous with the slow interchange of xanthates between dormant and active chains and is in agreement with the high experimental *Đ* values of current and past studies [32,47]. In comparison, d’Hooge et al. estimated a value of 0.44 for C_tr_ from conversion data upon polymer isolation, using dialysis and applying chain extension [33].

### 2.4. Acrylate Monomers

**Scheme 4:**
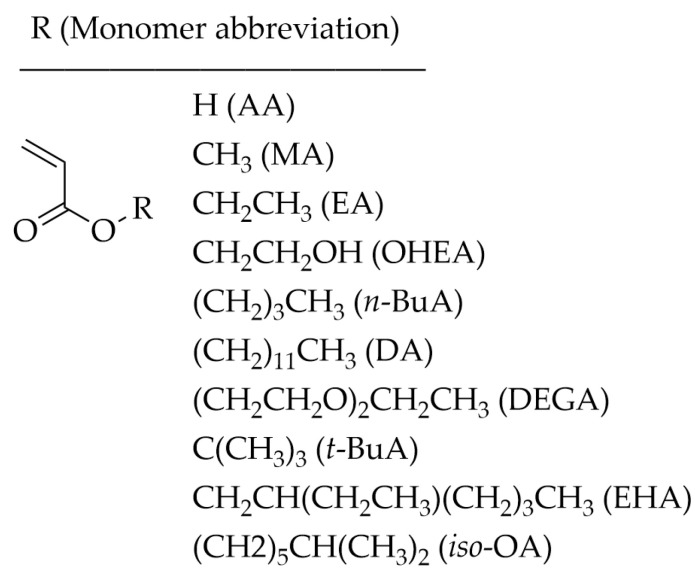
Acrylic monomers of the study.

XA1-mediated RAFT/MADIX polymerization of ten different acrylic monomers (Scheme 4) was investigated at 60 °C with XA1. Methyl acrylate (MA), 2-hydroxyethylacrylate (OHEA), dodecyl acrylate (DA), diethyleneglycol ethyl ether acrylate (DEGA), *t*-butyl acrylate (*t*-BuA), 2-ethytlhexylacrylaye (EHA), and isooctyl acrylate (*iso*-OA) were polymerized in bulk with monomer and XA1 concentrations which corresponded to a targeted *M_n_* of 10,000 g/mol. For ethyl acrylate (EA) and n-butyl acrylate (*n*-BuA), the chosen XA1 concentration was set so that *M_n,th_* was equal to 5000 g/mol. Acrylic (AA) was polymerized in a 50 wt.% solution, either in pure ethanol or ethanol/water (1:4.5). These two conditions are representative of protocols found in the literature for the synthesis of AA-based block copolymers with XA1 [34,48].

The evolution of *M_n_* and dispersities during polymerization of the series of acrylate monomers is presented in Figures S9 and S10. *M_n_* values for AA and HEA are not reported owing to irrelevant PEO calibration, with data strongly differing from those expected based on measured C_tr_^app^ values. The *M_n_* evolution profiles are very similar for all monomers of the series. A nearly linear increase in molar masses is observed, with higher values than expected for a controlled RAFT process during most of the polymerization. *M_n_* values get very close to *M_n,th_* in the final stages of polymerization, suggesting that XA1 has almost totally reacted at this stage. *Đ* values are in the 1.6–1.8 range towards the end of polymerization, except for DEGA for which *Đ* ranged from 1.8 to 2.2, presumably because of some contribution of irreversible chain transfer from CH_2_ in alpha to oxygen in ethylene oxide moieties.

C_tr_^app^ values are gathered in Table 2. The double log plots are available in Figures S11–S21. Values only vary by a factor of two with the nature of ester group: from 0.93 for EA to 1.89 for AA in water/ethanol. It seemed complex to us to discuss the effect of acrylic ester groups on *C_tr_* values, owing to little variation in k_p_ [49] and C_tr,_ not to mention the difficulty of accessing reliable k_p_ data for acrylates [50,51]. However, C_tr_^app^ for acrylates bearing secondary (EHA, iso-OA) or tertiary (*t*-BuA) carbons tend to be higher (1.45–1.77) than their linear counterparts (0.93–1.33). With *n*-BuA taken as an example, the found value is in agreement with the earlier reports of Smulders et al. (C_tr_^app^ = 1.62 at 50 °C for bulk conditions, using the Mayo method) [52]. Jacquin et al. reported a C_tr_^app^ value of 2.7 for DEGA using the double log plot method [34], which is roughly three times higher than the value we determined. However, these authors performed the reaction in ethanol when we used bulk conditions, which may contribute to the differences observed.

**Figure 1:**
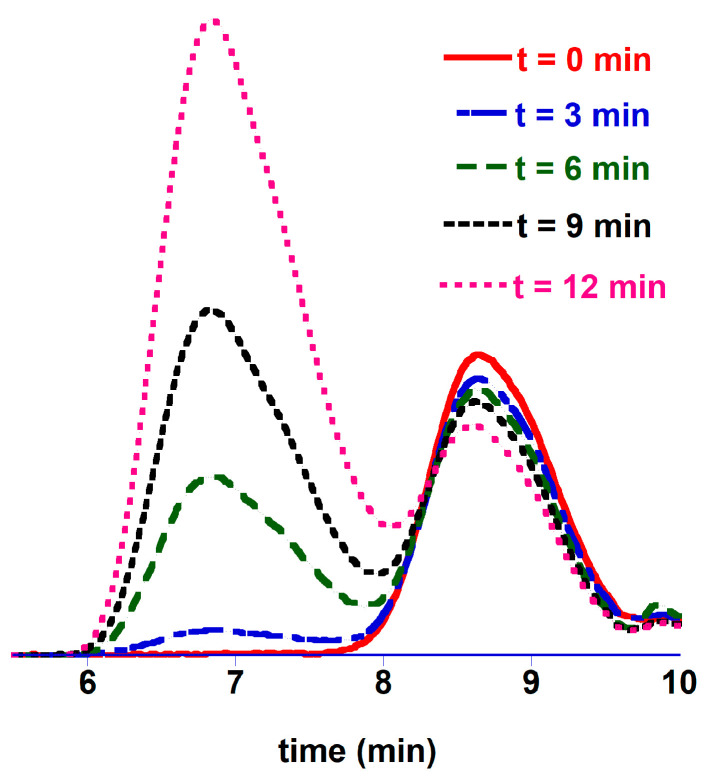
Overlay of SEC-RI chromatograms used to determine C_tr,PnX_ from the SEC-peak resolution method for the *n*-BuA/XA1 system at 60 °C in THF. [*n*-BuA]_0_ = 6.7 mol/L, [P(*n*-BuA)-XA_1_]_0_ = 2.6 × 10^−2^ mol/L, [AIBN]_0_ = 1.77 × 10^−2^ mol/L.

**Figure 2:**
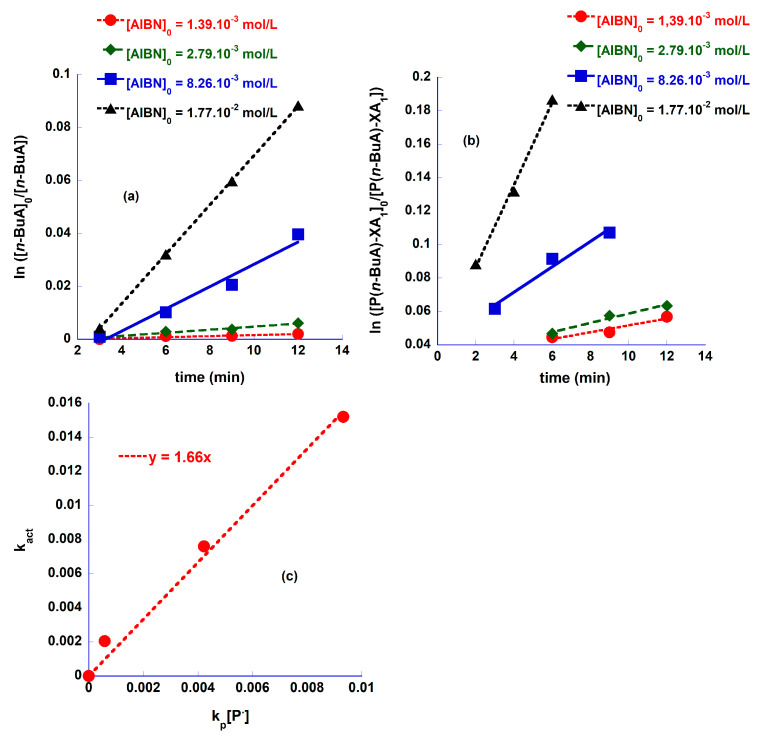
Determination of C_tr,PnXA1_ for *n*-butyl acrylate at 60 °C with AIBN (**a**) Plot of Ln([M]_0_/[M]) vs. time (**b**) Plot of Ln([S]_0_/[S]) vs. time (**c**) Plot of k_act_ vs. k_p_[P**^.^**].

Interchange transfer coefficients C_tr,PnXA1_ of 1.7 and 2.5 have been determined, respectively, for *n*-BuA and *t*-BuA homopolymerizations. Data are shown in Figure 1 and Figure 2 for *n*-BA and in Figure S22 for *t*-BA. For *n*-BA, this value is very close to C_tr_^app^ (1.33), which can be explained by similar structures of R• and P_n_• radicals after fragmentation (Scheme 2). The C_tr,PnXA1_ value is in excellent agreement with the observed dispersities at high conversions (*Đ*~1.6). Indeed, the kinetic analysis of Müller and co-workers for living/controlled polymerizations based on degenerative processes estimated that dispersity should tend towards 1 + 1/C_ex_ (~1.6 in our case) at the end of polymerization [41]. The same conclusions cannot be drawn with *t*-BuA because the experimental conversion points do not exceed 40%. Dispersities values neighboring 1.4 can be expected at the end of polymerization based on determined C_tr,PnXA1_.

### 2.5. Acrylamido Monomers

**Scheme 5:**
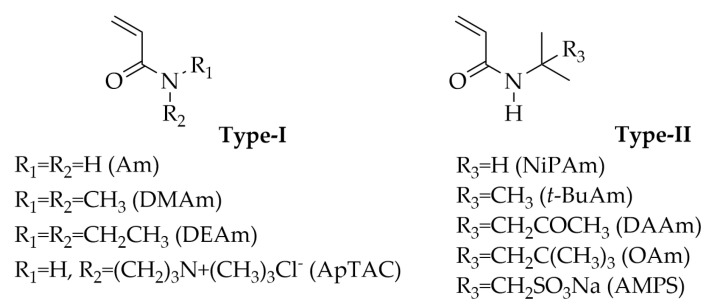
Acrylamide monomers of the study.

RAFT/MADIX polymerization of nine acrylamides (Scheme 5) was investigated at 60 °C with XA1. Type-I acrylamides contain either hydrogen or primary alkyl substituents on amide nitrogen. It includes acrylamide (Am), *N*,*N*-dimethylacrylamide (DMAm), *N*,*N*-diethylacrylamide (DEAm), and 3-acrylamidopropyltrimethylammonium chloride (ApTAC). *N*-isopropylacrylamide (NiPAm), *t*-butylacrylamide (*t*-BuAm), diacetone acrylamide (DAAm), *N*-*tert*-octylacrylamide (OAm), and sodium 2-acrylamido-2-methylpropane sulfonate (AMPS) are type-II monomers which bear both hydrogen and tertiary alkyl substituents.

The evolution of *M_n_* and *Đ* with conversion are presented in Figures S23 and S24 for Type-I and Type-II monomers, respectively. Only data for PApTAC and PAMPS are missing due to the lack of SEC conditions available for strong polyelectrolytes. A nearly linear increase of *M_n_* with conversion was obtained for PAm, in agreement with expected values for controlled polymerization. The corresponding dispersities decreased over time and equalled ~1.6 after 62% conversion (Figure S23). For DMAAm and DEAm, *M_n_* values were higher than those predicted for a fast consumption of XA1 and matched the expected values at about 30% conversion. Dispersities tended to increase with conversion, with *Đ* in the 1.15–1.25 range for DMAm and in the 1.4–1.8 range for DEAm (Figure S23). For Type-II monomers (Figure S24), all the polymers of the series exhibited a similar *M_n_* evolution profile, with increasing values with conversion which tended to be slightly higher than those calculated for a fast XA1 consumption. Dispersities tended to increase with conversion, in the 1.2–1.3 range at low conversion and in the 1.35–1.55 range after ~40% conversion. We attributed the observed increase in *Đ* with conversion for the series of hydrophobic polyacrylamides to irreversible polymer aggregation upon drying during sample purification. Indeed, Ganachaud et al. made similar observations after they obtained different SEC results in THF for PNiPAm prepared by RAFT depending on the mode of sample preparation [53]. Although SEC results were not used for the determination of transfer coefficients, the use of DMF/LiBr as eluent would have given more reliable molar mass data, as LiBr in DMF is known to screen ionic and polar group interactions in polymer chains [54].

From Table 3 and Table 4, it can be concluded that C_tr_^app^ values for acrylamides are slightly greater than those of styrenic and acrylate monomers and vary little according to the nature of the substituents: transfer coefficients to XA1 are in the 2.3–3.5 range for Type-I monomers and between 2.4 and 3.3 for Type-II monomers.

**Figure 3:**
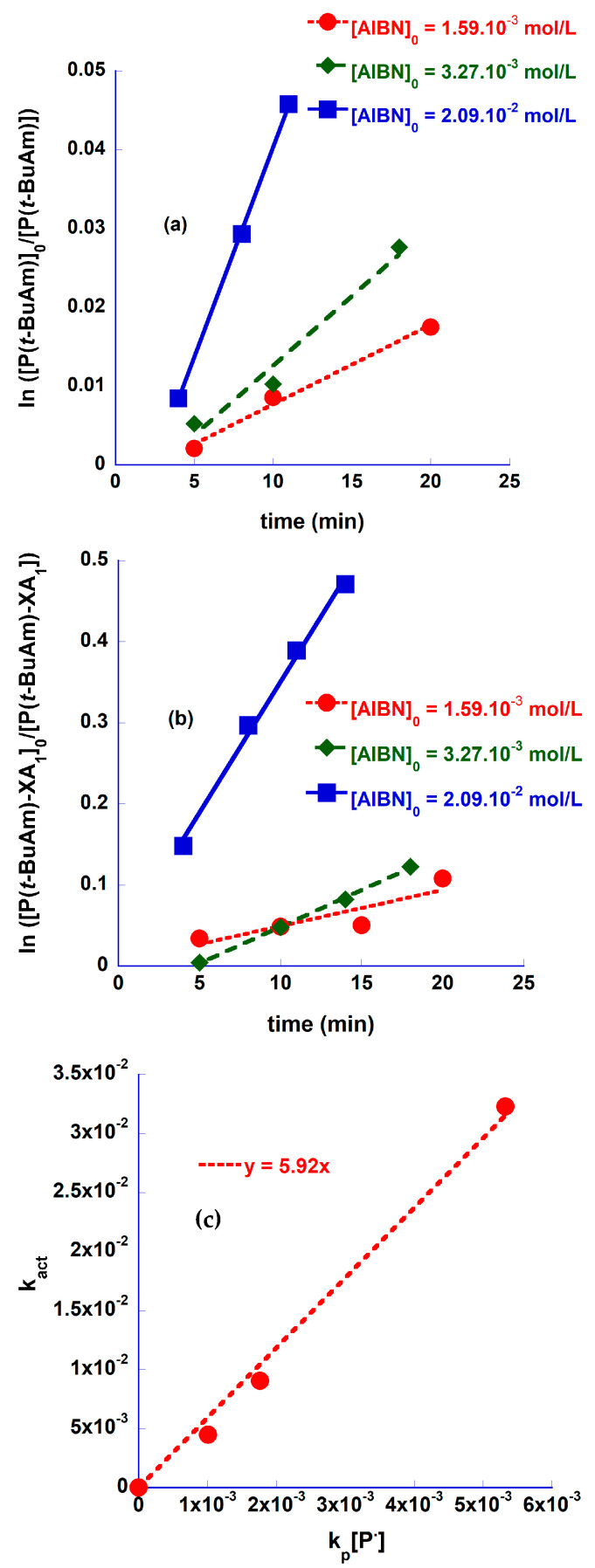
Determination of C_tr,PnXA1_ for the *t*-butyl acrylamide at 60 °C (**a**) Ln([M]_0_/[M]) vs. time. (**b**) Ln([S]_0_/[S]) vs. time. (**c**) Plot of k_act_ vs. k_p_[P**^.^**]. The leading coefficient gives C_tr,PnXA1_.

Interchange transfer coefficients C_tr,PnXA1_ were determined for Am (Table 3 and Figure S34) and *t*-BuAm (Table 4 and Figure 3) monomers, the SEC-peak resolution method giving values of 1.3 and 5.9, respectively. For Am, C_tr,PnXA1_ is lower than C_tr_^app^ (3.3), which can be explained by the fact that the methylpropionate group of XA1 is a better leaving group than the terminal acrylamide unit. In other terms, this reflects the relative stabilities or R• (pre-equilibrium, Scheme 2) and P_n_• (main equilibrium, Scheme 2) radicals. For *t*-BuAm, the trend is opposite with C_tr_^app^ of 2.4: in this case, the bulky *t*-BuAm macro-leaving group favors addition−fragmentation relative to the XA1 leaving group.

### 2.6. Past Works on Other Monomers

In order to gather as many elements as possible to establish an up-to-date reactivity map of Rhodixan^®^ A1, this section is a reminder of previous works on its chain transfer kinetics. Dufils et al. [18] studied the RAFT/MADIX polymerization of vinyl acetate (VAc) with various xanthates including XA1, which was shown to be highly reactive in bulk conditions at 60 °C with a marked initialization period (formation of XA1:VAc 1:1 adduct prior to polymerization), rendering the determination of C_tr_^app^ impossible. The formed monoadduct was isolated and used as a new RAFT/MADIX agent in VAc polymerization. A high C_tr_^app^ value of 25.7 was determined from the double log plot method. As a first approximation, this value can be assimilated to C_tr,PnXA1,_ owing to similar structures of monoadduct and PVAc-XA1. This was in agreement with the well-established fast interchain transfer of *O*-ethyl xanthate groups in vinyl ester polymerization leading to low dispersity polymers [19]. Diallyldimethylammonium chloride (DADMAC), a monomer used industrially for the production of cationic polyelectrolytes, was polymerized by RAFT/MADIX with XA1 at 50 °C, initiated by V-50 in water/EtOH [27]. A high value of C_tr_^app^ = 18.8 was determined, which supported the excellent control of molar masses throughout polymerization. In contrast, *Đ* values were unexpectedly high (2.0–2.2), suggesting a slow xanthate group transfer between dormant and active chains. This was confirmed by the low measured value of C_tr,PnXA1_ = 1.5.

## 3. Materials and Methods

### 3.1. Materials

Methyl acrylate (Burlington, MA, USA, Sigma-Aldrich, 99%), ethyl acrylate (EA, Sigma-Aldrich, ≥99.5%), 2-hydroxyethyl acrylate (OHEA, Sigma-Aldrich, 96%), *n*-butyl acrylate (*n*-BuA, Sigma-Aldrich, ≥99%), *tert*-butyl acrylate (*t*-BuA, Sigma-Aldrich, 98%), 2-ethylhexyl acrylate (EHA, Sigma-Aldrich, 98%), and styrene (St, Sigma-Aldrich, ≥99%) were freed from the inhibitor (MEHQ) by passing through a column of activated basic alumina. Isooctyl acrylate (*iso*-OA, Sigma Aldrich, >90%), dodecyl acrylate (DA, Sigma-Aldrich, 90%), diethyleneglycol ethyl ether acrylate (DEGA, Sigma Aldrich, technical grade), 4-methylstyrene (*p*-MSt, Sigma-Aldrich, 96%), 4-methoxystyrene (*p*-MeOSt, Sigma-Aldrich, for synthesis), 4-acetoxystyrene (*p*-AcOSt, Sigma-Aldrich, 96%), and 4-(chloromethyl)styrene (*p*-ClMSt, Sigma-Aldrich, ≥90%) were purified by distillation under vacuum before use. Sodium 4-vinylbenzene sulfonate (*p*-StSO_3_, Sigma-Aldrich, ≥90%), acrylic acid (AA, Sigma-Aldrich, 99%), acrylamide (Am, Rhodia, La Defence, France, 50 wt.% in water), *N*,*N*-dimethylacrylamide (DMAm, Sigma-Aldrich, 99%), *N*,*N*-diethylacrylamide (DEAm, Sigma-Aldrich, 99%), 3-acrylamidopropyltrimethylammonium chloride (ApTAc, Aldrich, 75 wt.% in water), *N*-isopropylacrylamide (NiPAm, Sigma-Aldrich, ≥99%), *tert*-butylacrylamide (*t*-BuAm, Sigma-Aldrich, 97%), diacetone acrylamide (DAAm, Sigma-Aldrich, 99%), *N*-*tert*-octylacrylamide (OAm, Merck, Molsheim, France, 95%), and sodium 2-acrylamido-2-methylpropanesulfonate (AMPS, Sigma-Aldrich, 50 wt.% in water) were used as received. 4,4′-azobis(4-cyanovaleric) acid (ACVA, Sigma-Aldrich, ≥98%), 2,2′-Azobis(2-methylpropionamidine) dihydrochloride (V-50, Sigma-Aldrich, 97%), ethanol (Normapur, Strasbourg, France), and *O*-ethyl-*S*-(1-methoxycarbonyl)ethyldithiocarbonate (XA1, Rhodia) were used as received. Azobis(isobutyronitrile) (AIBN, Janssen, Geel, Belgium) was recrystallized twice from methanol prior to use.

### 3.2. Polymerizations

RAFT/MADIX polymerization of hydrophobic monomers in bulk. A typical polymerization was realized as follows: XA1 (170 mg, 0.816 mmol), St (8 g, 76.8 mmol), and AIBN (12 mg, 0.0761 mmol) are placed in a 25 mL-Schlenk flask under stirring for 10 min. The solution is then divided into eight glass tubes. The tubes were degassed by four vacuum-freeze–thaw cycles and then sealed under vacuum. Polymerizations were performed by placing the tubes in an oil bath at 60 °C and the tubes were removed after a recorded time interval. The samples were finally quenched with liquid nitrogen.

RAFT/MADIX polymerization in ethanol. A typical polymerization was realized as follows: XA1 (170 mg, 0.816 mmol), tert-butyl acrylamide (8 g, 63 mmol), ethanol (8 g, 174 mmol), and AIBN (12 mg, 0.0761 mmol) are placed in a 25 mL-Schlenk tube under stirring for 10 min. The solution is then divided into eight glass tubes. The tubes were degassed by four vacuum-freeze–thaw cycles and then sealed under vacuum. Polymerizations were performed by placing the tubes in an oil bath at 60 °C and the tubes were removed after a recorded time interval. The samples were finally quenched with liquid nitrogen.

RAFT/MADIX polymerization in water/ethanol. A typical polymerization was realized as follows: XA1 (83 mg, 0.4 mmol), AMPS (8.5 g of a 50 wt.% solution in water, 18 mmol), ethanol (1.25 g, 28 mmol), distilled water (1.6 g, 91 mmol), and ACVA (23.5 mg, 0.0834 mmol) are placed in a two-neck flask under stirring for 10 min. The reaction mixture was degassed by bubbling argon for 30 min. Then, the flask is placed in an oil bath at 60 °C. Samples are withdrawn after a recorded time interval.

Synthesis of P(St) macro-RAFT/MADIX agent (PSt-XA1) for the determination of C_tr,PnXA1_. XA1 (2.41 g, 11.5 mmol), St (19.0 g, 182 mmol), and AIBN (6.2 mg, 0.38 mmol) were placed in a 50 mL Schlenk flask. The polymerization mixture was degassed by purging with argon for 20 min and the Schlenk flask was placed in an oil bath at 60 °C for eight hours. Then, the polymer was purified using precipitation in cold methanol. M_n_ = 3100 g/mol (M_n,th_ = 2000 g/mol).

Synthesis of P(n-BuA) macro-RAFT/MADIX agent (P(n-BuA)-XA1) for the determination of C_tr,PnXA1_. XA1 (3.05 g, 14.6 mmol), n-BuA (19.0 g, 182 mmol), and AIBN (38.6 mg, 0.24 mmol) were placed in a 60 mL Schlenk flask. The polymerization mixture was degassed by purging with argon for 20 min and the Schlenk flask was placed in an oil bath at 60 °C for eight hours. Then, the polymer was purified using precipitation. M_n_ = 2000 g/mol (M_n,th_ = 1900 g/mol).

Synthesis of P(*t*-BuA) macro-RAFT/MADIX agent (P(*t*-BuA)-XA1) for the determination of C_tr,PnXA1_. XA1 (3.05 g, 14.6 mmol), *t*-BuA (19.0 g, 182 mmol), and AIBN (38.6 mg, 0.24 mmol) were placed in a 60 mL Schlenk flask. The polymerization mixture was degassed by purging with argon for 20 min and the Schlenk flask was placed in an oil bath at 60 °C for eight hours. Then, the polymer was purified with precipitation. M_n_ = 1900 g/mol (M_n,th_ = 1800 g/mol).

Synthesis of P(Am) macro-RAFT/MADIX agent (PAm-XA1) for the determination of C_tr,PnXA1_. XA1 (1.16 g, 5.6 mmol), Am (20 g of a 50 wt.% solution in water, 141 mmol), ethanol (5 g, 111 mmol), distilled water (12.5 g, 694 mmol), and V-50 (23.5 mg, 0.087 mmol) were placed in a 50 mL Schlenk flask. After the polymerization mixture was degassed by purging with argon for 20 min, the Schlenk flask was placed in an oil bath at 60 °C for eight hours. Then, the polymer was purified with precipitation. M_n_ = 2100 g/mol (M_n,th_ = 2000 g/mol).

Synthesis of P(*t*-BuAm) macro-RAFT/MADIX agent (P(*t*-BuAm)-XA1) for the determination of C_tr,PnXA1_. XA1 (1.3 g, 6.3 mmol), *t*-BuAm (8 g, 63 mmol), ethanol (24 g, 533 mmol), and AIBN (30 mg, 0.18 mmol) were placed in a 50 mL Schlenk flask. After the polymerization mixture was degassed by purging with argon for 20 min, the Schlenk flask was placed in an oil bath at 60 °C for eight hours. Then, the polymer was purified with precipitation. M_n_ = 1500 g/mol (M_n,th_ =1400 g/mol).

General procedure for the determination of C_tr_^app^.

The evolution of monomer and XA1 concentrations during polymerization was followed by HPLC. The linear regression for Ln[XA1] as a function of Ln[M] gives C_tr_^app^ as a leading coefficient. The associated uncertainty was calculated from the systematic error made on the value of the leading coefficient of the linear regression.

### 3.3. General Procedure for the Determination of the Interchain Transfer Coefficient C_tr,PnXA1_

A typical protocol is given as follows, with P(*n*-BA) taken as an example: for chain extension experiment, P_0_(*n*-BA)-XA1 (0.228 g, 0.114 mmol), *n*-BA (4 g, 31 mmol), and AIBN (8 mg, 0.029 mmol) were placed in a 15 mL Schlenk flask. The polymerization mixture was divided into four glass tubes. The tubes were degassed by four vacuum-freeze–thaw cycles and then sealed under vacuum. Polymerizations were performed by placing the tubes in an oil bath at 60 °C, then removing them after a recorded time interval. The samples were quenched by liquid nitrogen, then diluted in THF to a known concentration and analyzed using SEC. As shown in Figure 1, the total area under each curve relative to that of the t = 0 curve allows the direct determination of the monomer concentration converted to polymer and consequently k_p_[P^.^] (Figure 2a). k_act_ is calculated by comparing the area under the chromatogram of P_0_(*n*-BA)-XA1 at time t relative to that at t = 0 after a peak deconvolution (Figure 2b). When this methodology is repeated for different initial AIBN concentrations, different {k_act_; k_p_[P^.^]} set of values are obtained. The linear regression for k_act_ as a function of k_p_[P^.^] gives C_tr,Pn-XA1_ as leading coefficient (Figure 2c). k_d_ (intercept) has been set to zero as this reflects past results of Fukuda and co-workers for which exclusive RAFT mechanism with no contribution of thermally induced reversible activation−deactivation was evidenced [55]. The associated uncertainty was calculated from the systematic error made on the value of the leading coefficient of the linear regression.

### 3.4. Instrumentation

^1^H-NMR were recorded on Bruker (Karlsruhe, Germany) AMX 300. For all molecular weight analysis, a Waters modular system, comprising an autoinjector, a Shodex 5.0 µm bead-size guard column (50 × 7.5 mm), followed by a linear Waters colums (10^4^ Å), and a differential refractive index detector using THF as the eluent at 30 °C with a flow rate of 1 mL·min^−1^ was used. This system was calibrated using narrow PSt standards ranging from 540 to 75,000 g·mol^−1^. Molar masses were obtained from PS calibration and recalculated from Mark–Houwink–Sakurada parameters when available (for eight out of ten homopolymers [56,57,58,59,60,61], see Table S1 for details). For the molecular weight analysis of hydrophilic polymers, a Waters modular system, comprising a manual injector, a Shodex OHPak SB-806M HQ (Showa Denko, Tokyo, Japan) guard column followed by three OHPak SB-806M HQ colums, a UV detector and a differential refractive index detector using H_2_O with NaNO_3_ (0.1 N) and NaN_3_ (100 ppm) as the eluent at 25 °C with a flow rate of 1 mL·min^−1^ was used. This system was calibrated using narrow PEO standards ranging from 1080 to 634,000 g·mol^−1^. Average molar masses for polyacrylamide are recalculated using Mark–Houwink–Sakurada parameters of POE [62] and PAm [63] (Table S1). For HPLC measurements, an Alliance modular system, comprising an autoinjector, a Zorbax column, and a UV detector (λ = 210 and 280 nm simultaneously) with a flow rate of 1 mL/min was used. Depending on the polarity of the monomer used, different elution methods were used with CH_3_CN/H_2_O mixtures.

## 4. Conclusions

**Scheme 6:**
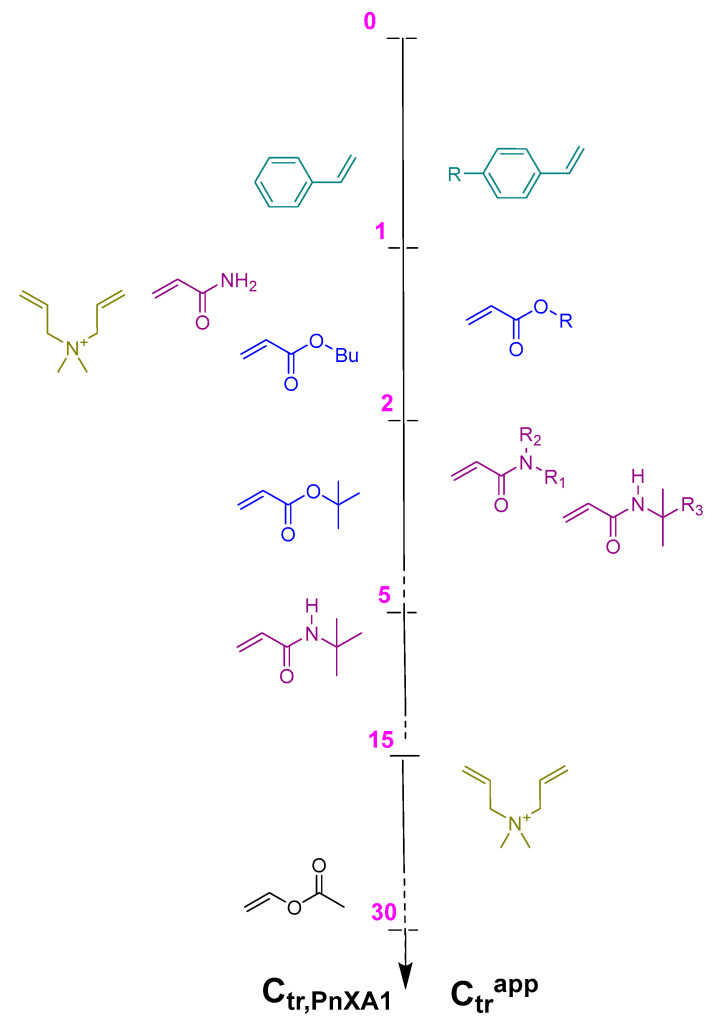
Reactivity scale of Rhodixan^®^ A1 in RAFT/MADIX polymerization of selected monomers.

The proposed kinetic study of chain transfer to XA1 and XA1-terminated polymers in radical polymerization has made it possible to establish a reactivity scale with respect to a wide range of monomers of interest (Scheme 6). It was established that RAFT/MADIX polymerization of styrene and derivatives is poorly controlled whatever the *p*-substituent. With C_tr_^app^ < 1, a significant amount of XA1 is left unreacted even at high monomer conversions. Polymer purification is mandatory prior to block copolymerization in order to avoid contamination with block B-type homopolymer. A way to get around this constraint is to force XA1 to react via semi-continuous introduction of the monomer. This approach is especially effective in emulsion polymerization, where the instantaneous monomer concentration can be kept low [64,65]. High dispersity block copolymers with good purity can be accessed in this way [64]. C_tr_^app^ are comprised between 0.89 and 1.89 for acrylates, and between 2.3 and 3.8 for acrylamides. In a practical way, as it is relatively easy to carry out the polymerization of these monomers until they are fully consumed, it becomes possible to prepare block copolymers either with conjugated of unconjugated monomers as second block, or without intermediate purification after first block synthesis. This can be a valuable asset for the development of block copolymer libraries using high-throughput screening methods for syntheses using parallel reactors [66,67] or flow chemistry [68]. By varying the combinations of the numerous conjugated and unconjugated monomers compatible with the reactivity of XA1, a plethora of copolymers of controlled chain length and architecture (block, gradient, star-like copolymers, nanogels, etc.) can be envisioned. These features undeniably make RAFT/MADIX one of the most robust technologies for advanced polymer synthesis.

## Figures and Tables

**Table 1:** Transfer coefficients to XA1 for styrenic monomers in bulk at 60 °C.

R	C_tr_^app^	C_tr,Pn-XA1_
H	0.58 (0.23) ^b^	0.64 (0.18) ^b^
CH_3_	0.85 (0.20)	
OCH_3_	1.01 (0.07)	
OCOCH_3_	0.88 (0.10)	
CH_2_Cl	0.83 (0.13)	
SO_3_Na ^a^	0.61 (0.17)	

^a^ in 50 wt.% water/ethanol (4.5:1); ^b^ calculated error determined by the least squares method.

**Table 2:** Transfer coefficients to XA1 for acrylate monomers in bulk at 60 °C.

R	C_tr_^app^	C_tr,Pn-XA1_
H ^a^	1.89 (0.23) ^c^	
H ^b^	1.30 (0.14)	
CH_3_	0.97 (0.16)	
CH_2_CH_3_	0.93 (0.12)	
CH_2_CH_2_OH	0.98 (0.30)	
(CH_2_)_3_CH_3_	1.33 (0.27)	1.7 (0.2) ^b^
(CH_2_)_11_CH_3_	1.01 (0.29)	
(CH_2_CH_2_O)_2_C_2_H_5_	0.94 (0.03)	
C(CH_3_)_3_	1.66 (0.38)	2.5 (0.2)
CH_2_CH(CH_2_CH_3_)(CH_2_)_3_CH_3_	1.45 (0.36)	
(CH_2_)_5_CH(CH_3_)_2_	1.77 (0.11)	

^a^ water/ethanol mixture (4.5:1, 50 wt.%); ^b^ ethanol (50 wt.%); ^c^ calculated error determined by the least squares method.

**Table 3:** Transfer coefficients to XA1 for Type-I acrylamides at 60 °C.

R_1_	R_2_	C_tr_^app^	C_tr,Pn-XA1_
H	H ^a^	3.5 (0.3) ^c^	1.3 (0.2) ^b^
H	(CH_2_)_4_N^+^(CH_3_)_3_Cl^− a^	2.8 (0.4)	
CH_3_	CH_3_^b^	2.3 (0.2)	
CH_2_CH_3_	CH_2_CH_3_^b^	2.6 (0.9)	

^a^ water/ethanol (4.5:1, 50 wt.%), ^b^ ethanol (50 wt.%), T = 60 °C, ^c^ calculated error determined by the least squares method.

**Table 4:** Transfer coefficients to XA1 for Type-II acrylamides at 60 °C.

R_3_	C_tr_^app^	C_tr,Pn-XA1_
H ^b^	3.3 (0.5) ^c^	
CH_3_^b^	2.4 (0.3)	5.9 (0.4) ^b^
CH_2_(CO)CH_3_^b^	3.0 (0.9)	
CH_2_C(CH_3_)_3_^b^	3.8 (0.2)	
CH_2_SO_3_Na ^a^	2.6 (0.1)	

^a^ water/ethanol (4.5:1, 50 wt.%), ^b^ ethanol (50 wt.%), T = 60 °C, ^c^ calculated error determined by the least squares method.

## Data Availability

The raw data supporting the conclusions of this article will be made available by the authors on request.

## Supplementary Materials

The following supporting information can be downloaded at: https://www.mdpi.com/article/10.3390/molecules29246004/s1, Figures S1, S9, S10, S23 and S24: *M_n_* and *Đ* vs. conversion; Figures S2–S8, S11–S21 and S25–S33: Determination of C_tr_^app^ from double log plot of [XA1] vs. [Monomer]; Figures S8, S22 and S34: Determination of C_tr,PnXA1_ from SEC-peak resolution method; Table S1: C_tr_^app^ to XA1 for a series of acrylate monomers for two different initial XA1 concentrations; Table S2: Mark–Houwink–Sakurada parameters.
