# Supplementary material for: Chain Transfer Kinetics of Rhodixan® A1 RAFT/MADIX Agent
Source: Molecules. 2024 Dec 20;29(24):6004. doi: 10.3390/molecules29246004 (PMC11678032; doi:10.3390/molecules29246004)
Supplement: Supplementary file 1 [file molecules-29-06004-s001.zip › molecules-3319039-supplementary.pdf]

SUPPLEMENTARY MATERIAL

Chain Transfer Kinetics of Rhodixan A1 RAFT/MADIX agent

Mathias Destarac [1],* Aymeric Guinaudeau [1,2], Stéphane Mazières[1] and James Wilson [3]

[1] Laboratoire SOFTMAT, CNRS UMR 5623, University of Toulouse, University Toulouse III-Paul Sabatier, 118 route de Narbonne, F-31062 Toulouse Cedex, France

[2] Syensqo, Centre de Recherche et Innovation Lyon, 85 rue des Frères Perret, BP 62 69192 Saint Fons, France

[3] Syensqo, Centre de Recherche et Innovation Aubervilliers, 52 rue de la Haie Coq, 93308 Aubervilliers Cedex, France

* Correspondence: mathias.destarac@univ-tlse3.fr

Table S1. C_{tr}^{app} to XA1 for a series of acrylate monomers for two different initial XA1 concentrations (corresponding to $M_{n,th}$=10^4 and 2.10^4 g/mol).

R	C_{tr}^{app}	
	$M_{n,th} = 10^4$ g/mol	$M_{n,th} = 2.10^4$ g/mol
CH_3	**0.97** (0.16)[a]	**1.05** (0.36)
CH_2CH_3	**0.93** (0.12)	**0.94** (0.36)
$(CH_2)_3CH_3$	**1.33** (0.27)	**1.32** (0.25)
$C(CH_3)_3$	**1.66** (0.8)	**1.63** (0.76)
$CH_2CH(CH_2CH_3)(CH_2)_3CH_3$	**1.45** (0.36)	**1.41** (0.47)
$(CH_2)_5CH(CH_3)_2$	**1.77** (0.11)	**1.66** (0.21)
$(CH_2)_{11}CH_3$	**1.01** (0.29)	**0.97** (0.16)

[a] Calculated error determined by the least squares method

Table S2. Mark-Houwink-Sakurada parameters.

Polymer	*K* (*dL/g*)	*a*
Poly(methyl acrylate)[a] [1]	$1{,}95.10^{-4}$	0,660
Poly(ethyl acrylate)[a] [2]	$1{,}81.10^{-4}$	0,626
Poly(*n*-butyl acrylate)[a] [3]	$1{,}22.10^{-4}$	0,700
Poly(*t*-butyl acrylate)[a] [4]	$4{,}34.10^{-4}$	0,600
Poly(2-ethylhexyl acrylate)[a] [3]	$8{,}20.10^{-5}$	0,695
Poly(isooctyl acrylate)[a] [5]	$5{,}14.10^{-5}$	0,740
Poly(dodecyl acrylate)[a] [3]	$2{,}92.10^{-4}$	0,585
Poly(ethylene oxide)[b] [6]	7.10^{-5}	0.750
Poly(acrylamide)[b] [7]	$4.9.10^{-5}$	0.800

[a] in THF at 30 °C [b] in water at 25°C

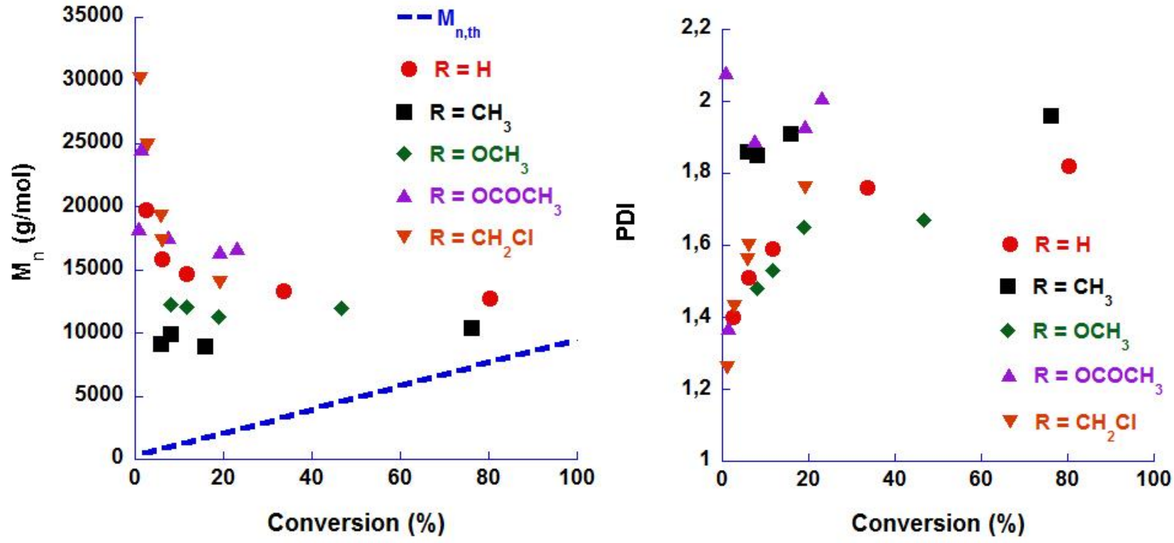

Figure S1. Evolution of (left) M_n and (right) $Đ$ versus conversion during RAFT/MADIX polymerization of *p*-substituted styrenic monomers in bulk at 60°C.

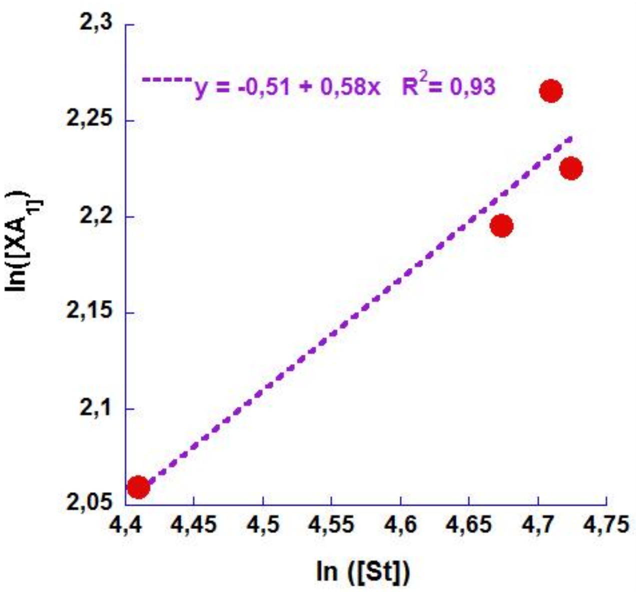

Figure S2. Double log plot of XA1 concentration vs St concentration. $[St]_0$ = 8.6 mol/L (bulk conditions), $[XA1]_0$ = 9.2.10^{-2} mol/L, $[AIBN]_0$ = 8.1.10^{-3} mol/L, T = 60°C.

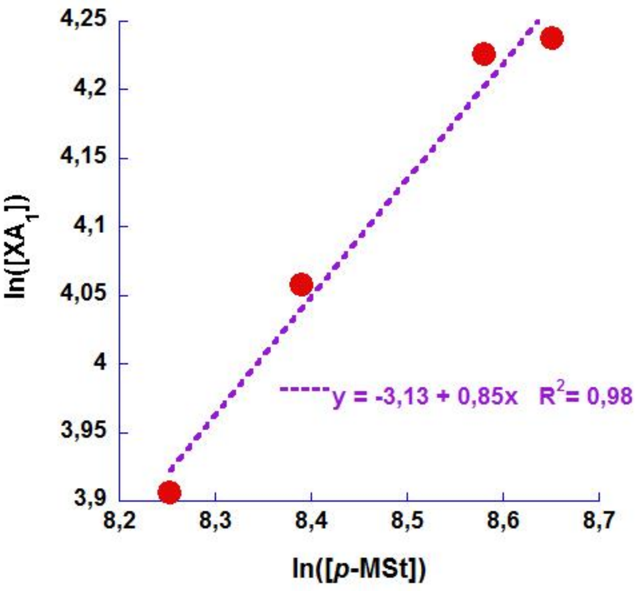

Figure S3. Double log plot of XA1 concentration vs *p*-MSt concentration. [*p*-MSt]0 = 7.5 mol/L (bulk conditions), [XA1]0 = 9.1.10-2 mol/L, [AIBN]0 = 8.1.10-3 mol/L, T = 60°C

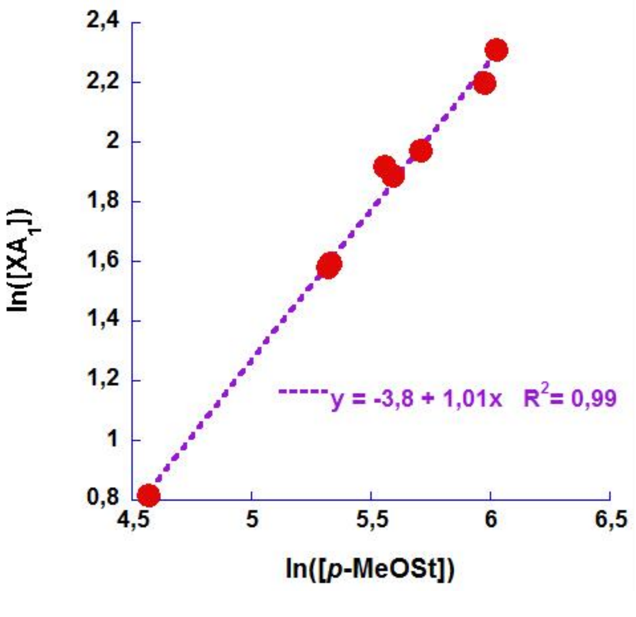

Figure S4. Double log plot of XA1 concentration vs *p*-MeOSt concentration. [*p*-MeOSt]0 = 7.3 mol/L (bulk conditions), [XA_1]0 = 9.2.10-2 mol/L, [AIBN]0 = 8.1.10-3 mol/L, T = 60°C.

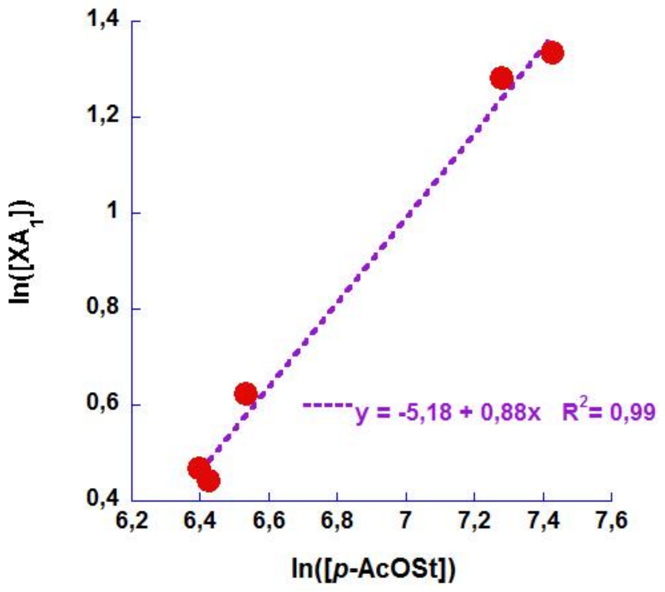

Figure S5. Double log plot of XA1 concentration vs *p*-AcOSt concentration. [*p*-AcOSt]$_0$ = 6.4 mol/L (bulk conditions), [XA_1]$_0$ = 9.1.10^{-2} mol/L, [AIBN]$_0$ = 8.1.10^{-3} mol/L, T = 60°C.

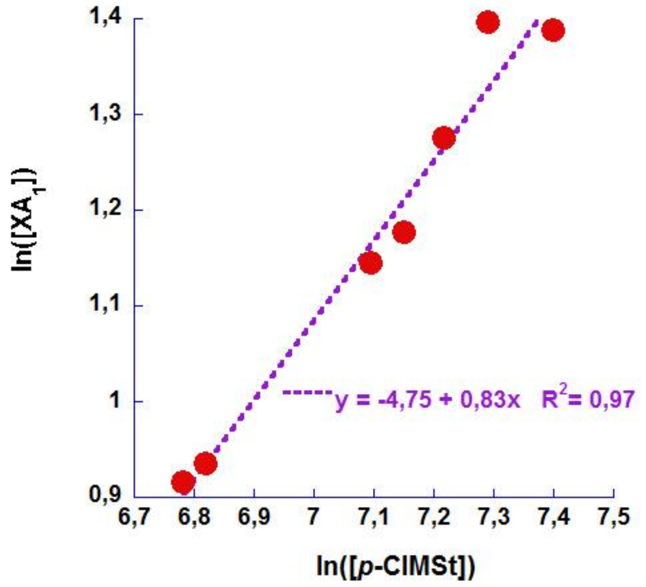

Figure S6. Double log plot of XA1 concentration vs *p*-ClMSt concentration. [*p*-ClMSt]$_0$ = 7.0 mol/L (bulk conditions), [XA_1]$_0$ = 9.8.10^{-2} mol/L, [AIBN]$_0$ = 8.1.10^{-3} mol/L, T = 60°C.

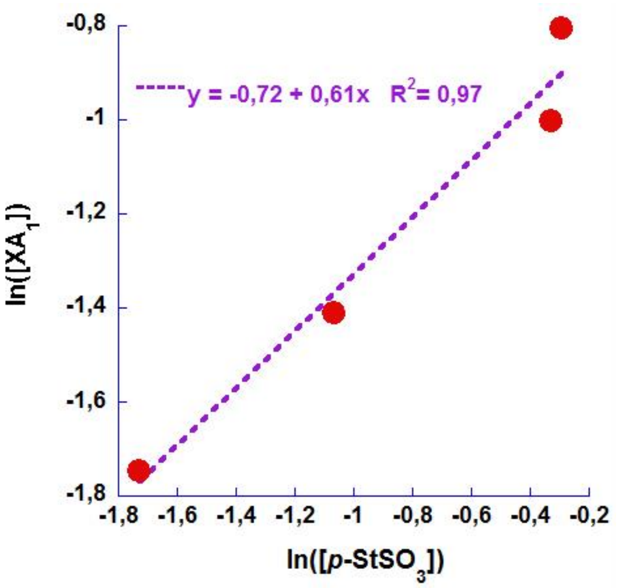

Figure S7. Double log plot of XA1 concentration vs *p*-$StSO_3$ concentration. [*p*-$StSO_3$]$_0$ = 0.66 mol/L, [XA1]$_0$ = 1.4.10^{-2} mol/L, [ACVA]$_0$ = 8.1.10^{-3} mol/L in 50 wt.% in water/ethanol (4.5 : 1). T = 60°C.

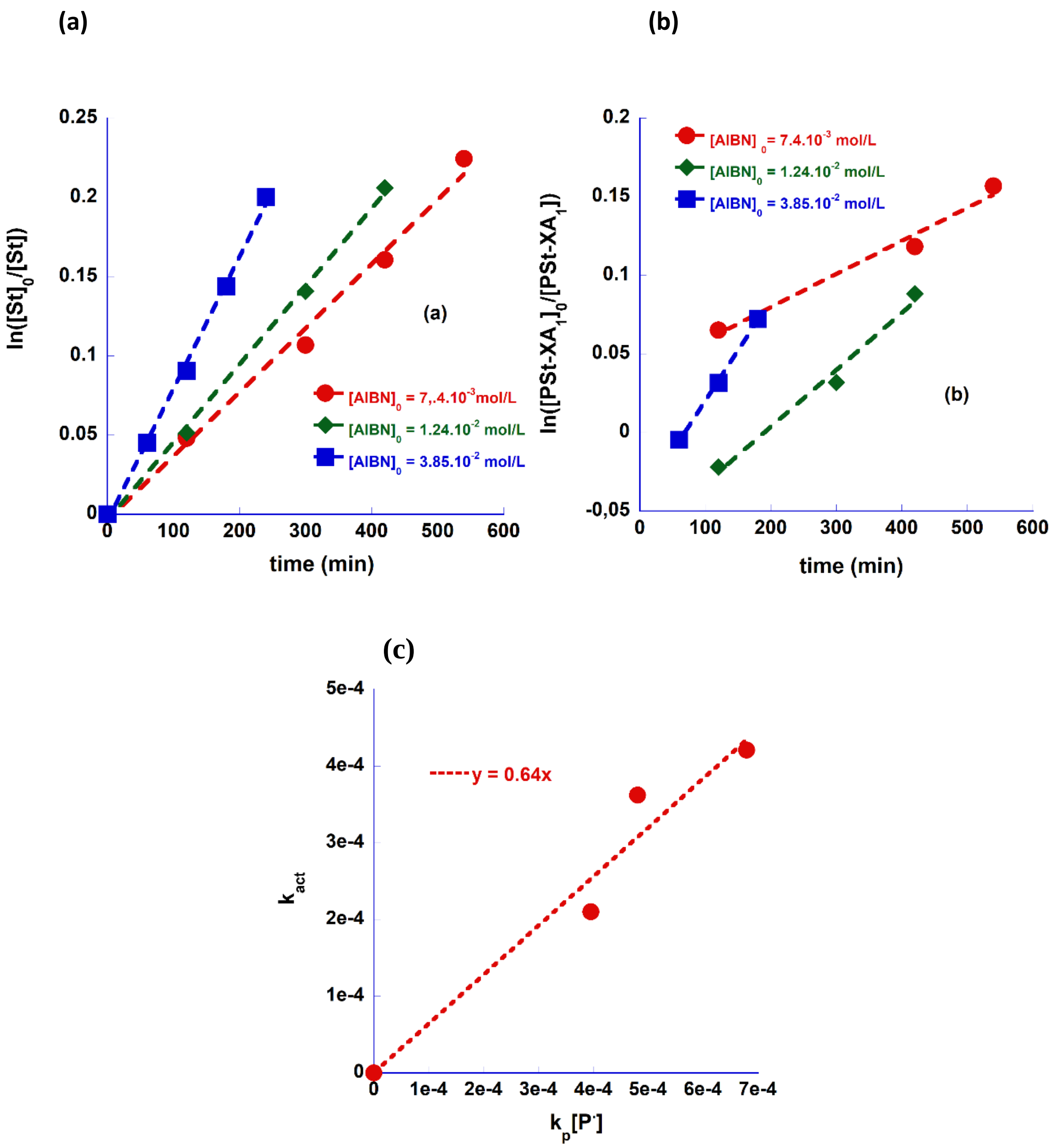

Figure S8. Determination of $C_{tr,PnXA1}$ for styrene at 60°C (a) Ln([M]$_0$/[M]) vs time (b) Ln([S]$_0$/[S]) vs time (c) Plot of k_{act} vs k_p[P·]. The leading coefficient gives $C_{tr,PnXA1}$.

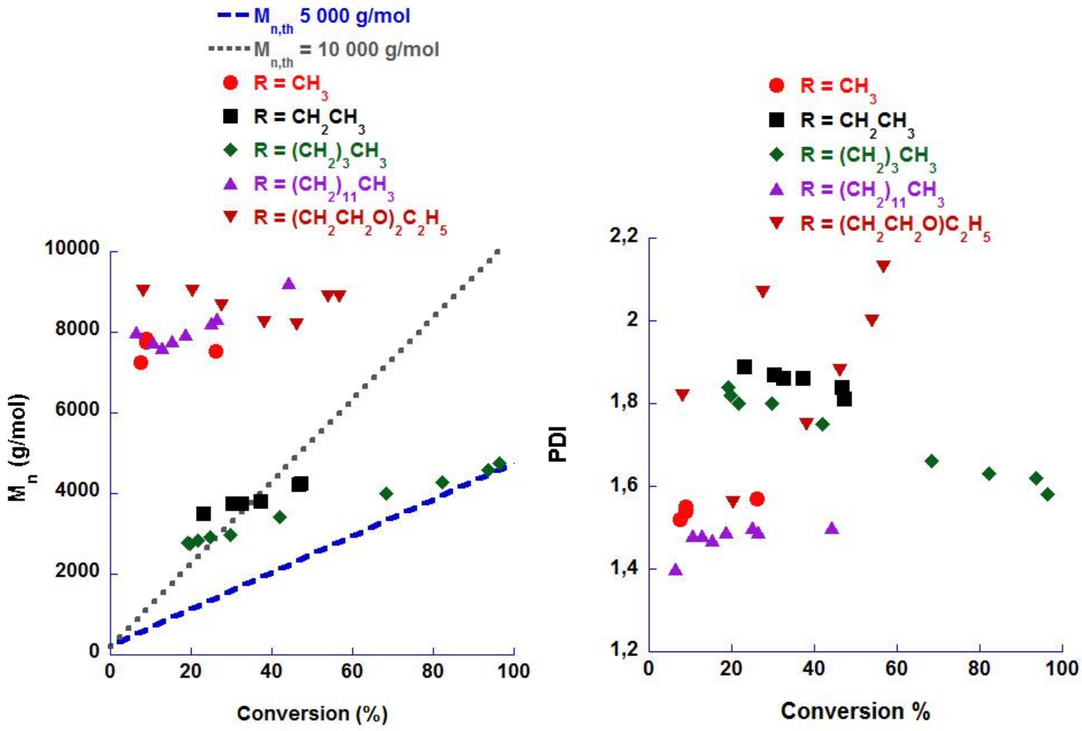

Figure S9. Evolution of M_n and *Đ* versus conversion during RAFT/MADIX bulk polymerization of acrylate monomers bearing linear R groups, T = 60°C.

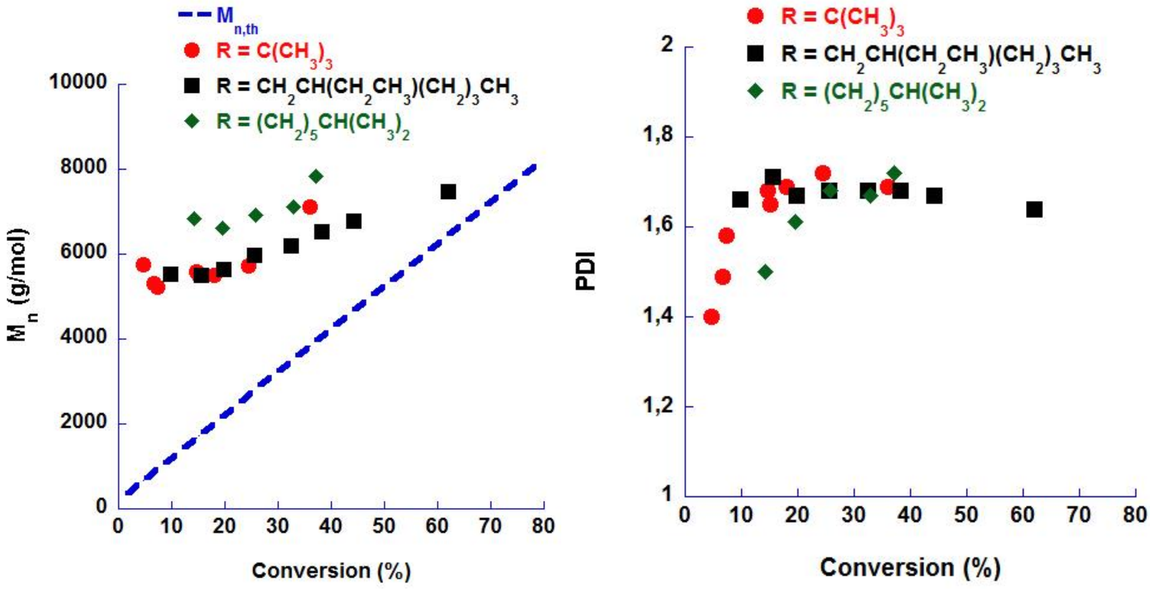

Figure S10. Evolution of *M_n* and *Đ* versus conversion during RAFT/MADIX bulk polymerization of acrylate monomers with R groups bearing secondary or tertiary carbons, T = 60°C.

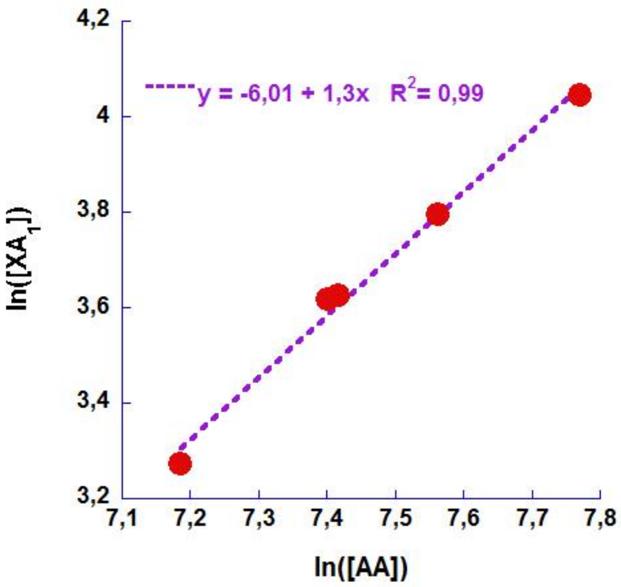

Figure S11. Double log plot of XA1 concentration vs AA concentration, $[AA]_0$ = 6.7 mol/L, $[XA1]_0$ = 5.10^{-2} mol/L, $[AIBN]_0$ = 4.10^{-3} mol/L, in 50 wt.% ethanol, T = 60°C.

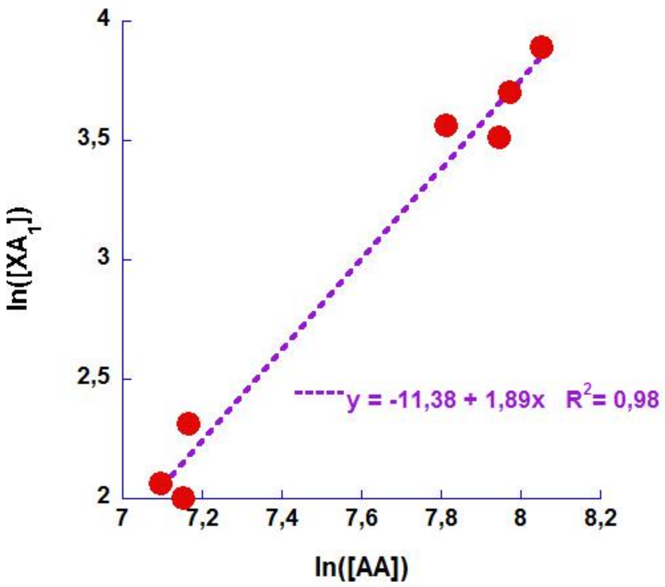

Figure S12. Double log plot of XA1 concentration vs AA concentration, $[AA]_0$ = 7.0 mol/L, $[XA1]_0$ = 5.2.10^{-2} mol/L, $[ACVA]_0$ = 4.0.10^{-3} mol/L, in 50 wt.% water / ethanol (4.5 : 1), T = 60°C.

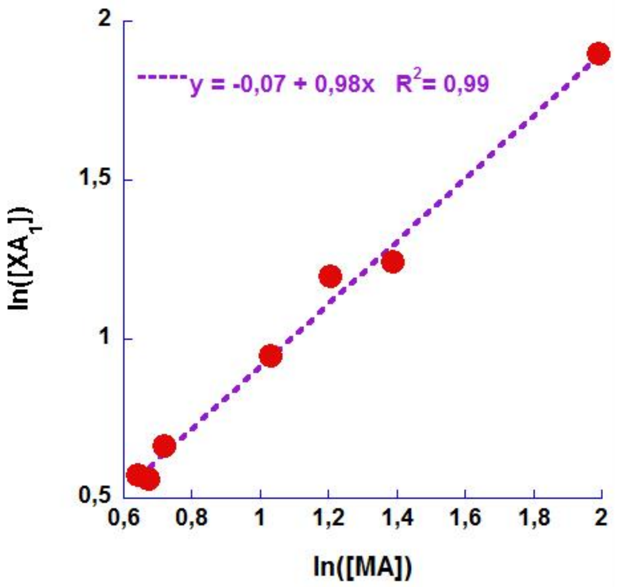

Figure S13. Double log plot of XA1 concentration vs MA concentration, $[MA]_0$ = 10.9 mol/L, $[XA1]_0$ = $9.2.10^{-2}$ mol/L, $[AIBN]_0$ = $8.1.10^{-3}$ mol/L, T = 60°C.

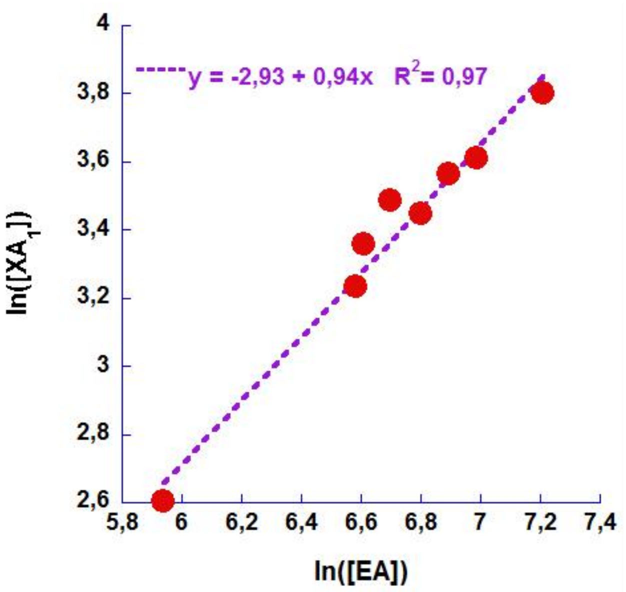

Figure S14. Double log plot of XA1 concentration vs EA concentration, $[EA]_0$ = 8.7 mol/L, $[XA1]_0$ = $1.9.10^{-1}$ mol/L, $[AIBN]_0$ = $8.1.10^{-3}$ mol/L, T = 60°C.

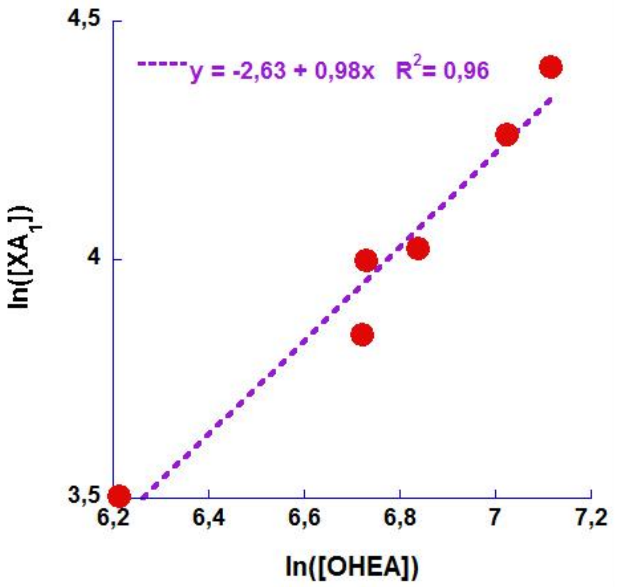

Figure S15. Double log plot of XA1 concentration vs OHEA concentration, $[OHEA]_0$ = 8,6 mol/L, $[XA1]_0 = 9.2.10^{-2}$ mol/L, $[AIBN]_0 = 8.1.10^{-3}$ mol/L, T = 60°C.

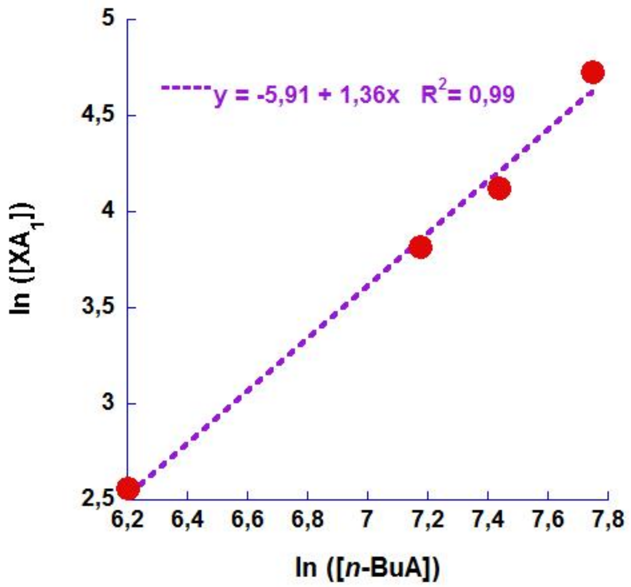

Figure S16. Double log plot of XA1 concentration vs *n*-BuA concentration, $[n\text{-BuA}]_0$ = 6.9 mol/L, $[XA1]_0 = 9.10^{-2}$ mol/L, $[AIBN]_0 = 8.1.10^{-3}$ mol/L, T = 60°C.

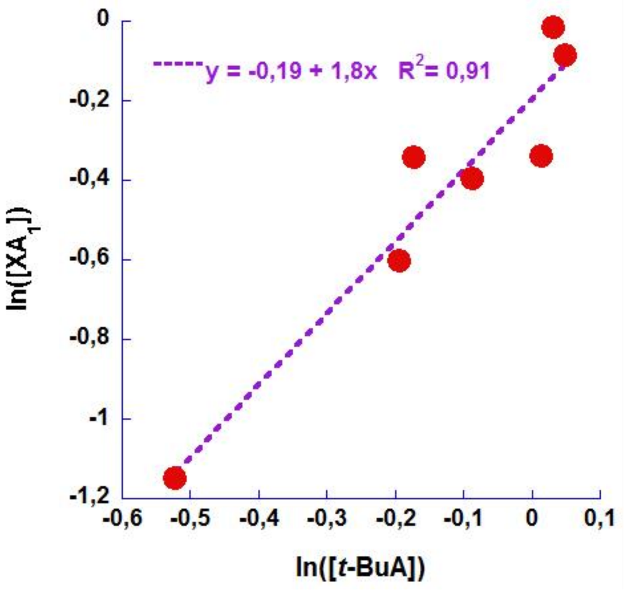

Figure 17. Double log plot of XA1 concentration vs *t*-BuA concentration, [*t*-BuA]$_0$ = 6.7 mol/L, [XA1]$_0$ = 9.1.10^{-2} mol/L, [AIBN]$_0$ = 8.1.10^{-3} mol/L, T = 60°C.

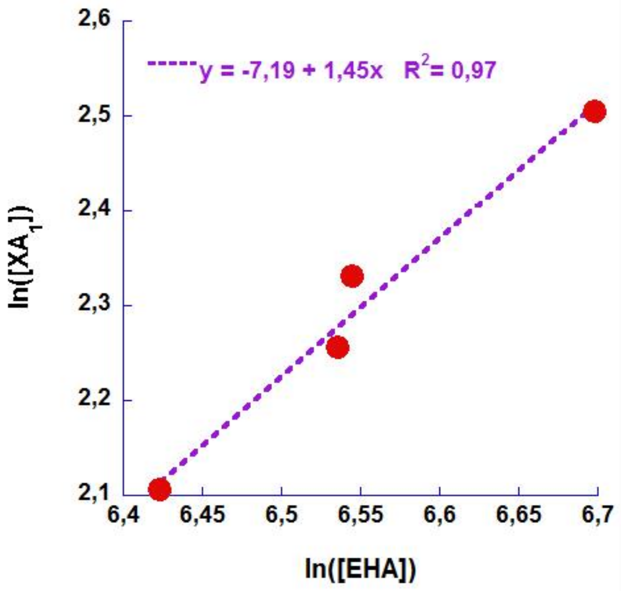

Figure S18. Double log plot of XA1 concentration vs EHA concentration, [EHA]$_0$ = 4.7 mol/L, [XA1]$_0$ = 1.2.10^{-1} mol/L, [AIBN]$_0$ = 8.10^{-3} mol/L, T = 60°C.

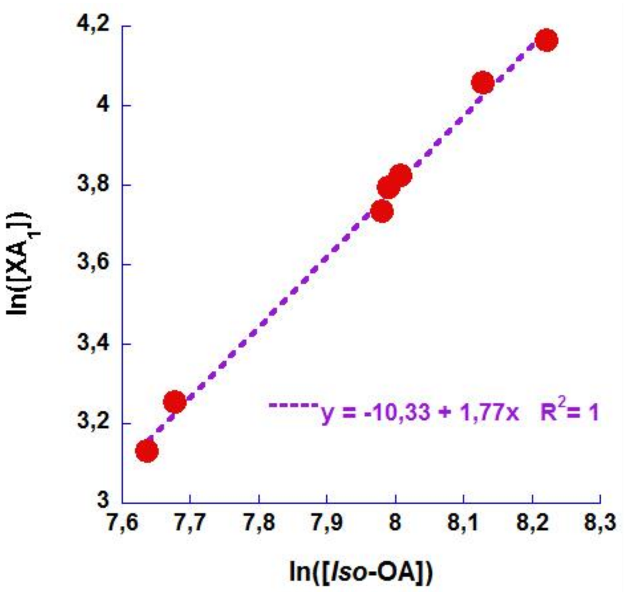

Figure S19. Double log plot of XA1 concentration vs *Iso*-OA concentration, $[Iso\text{-}OA]_0 = 4.7$ mol/L, $[XA1]_0 = 8.7.10^{-2}$ mol/L, $[AIBN]_0 = 8.1.10^{-3}$ mol/L, T = 60°C.

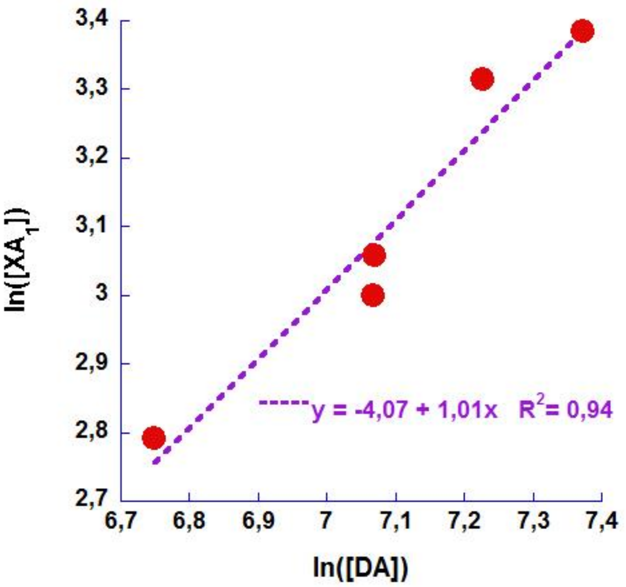

Figure S20. Double log plot of XA1 concentration vs DA concentration, $[DA]_0 = 3.6$ mol/L, $[XA1]_0 = 9.3.10^{-2}$ mol/L, $[AIBN]_0 = 8.5.10^{-3}$ mol/L, T = 60°C.

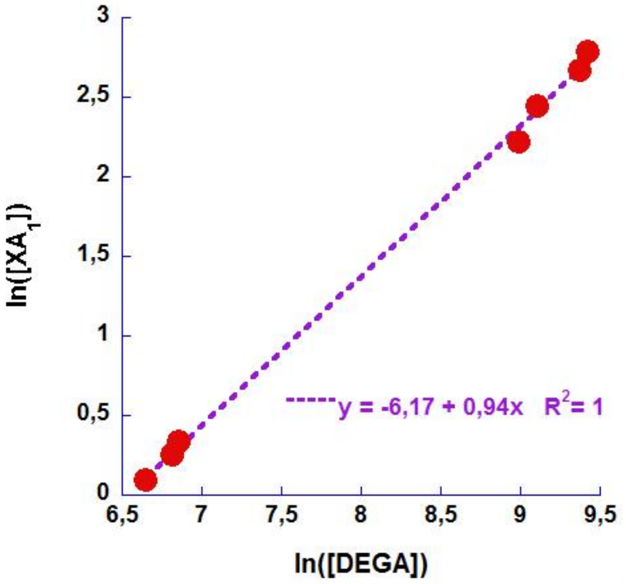

Figure S21. Double log plot of XA1 concentration vs DEGA concentration, $[DEGA]_0$ = 5.3 mol/L, $[XA_1]_0 = 1.10^{-1}$ mol/L, $[AIBN]_0 = 8.1.10^{-3}$ mol/L, T = 60°C.

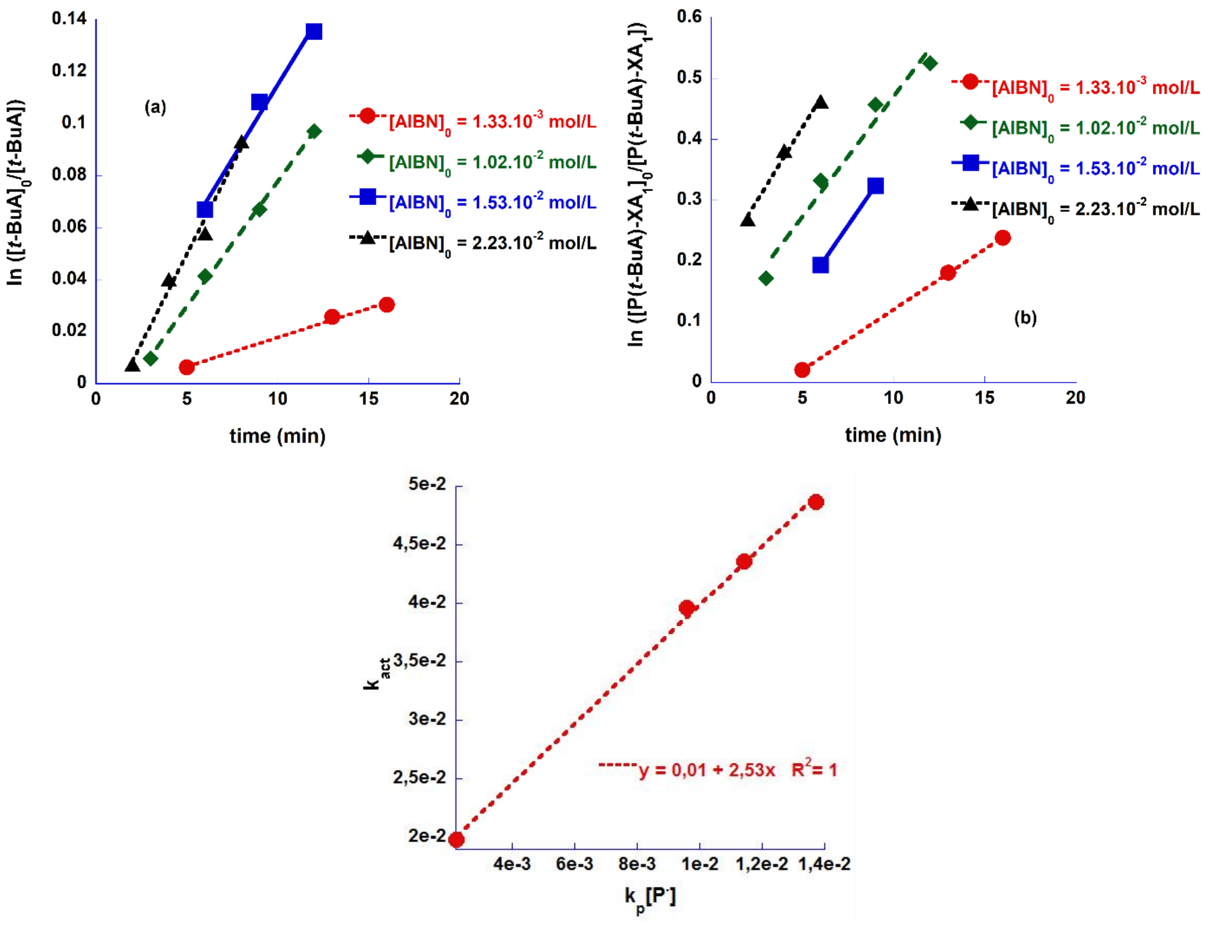

Figure S22. Determination of $C_{tr,PnXA1}$ for *t*-butyl acrylate at 60°C (a) $Ln([M]_0/[M])$ vs time (b) $Ln([S]_0/[S])$ vs time (c) Plot of k_{act} vs $k_p[P\cdot]$. The leading coefficient gives $C_{tr,PnXA1}$.

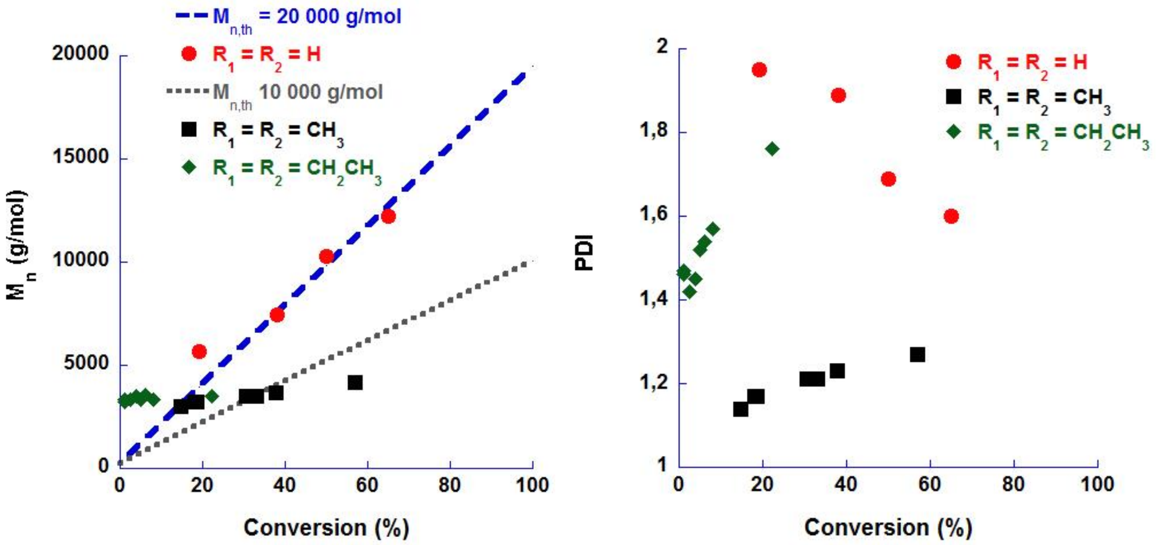

Figure S23. Evolution of M_n and PDI versus conversion during RAFT/MADIX polymerization of Type I-acrylamides at 60°C. $M_{n,th}$= 20 000 g/mol for Am (●) in 25 wt.% water / ethanol (4.5 : 1), $M_{n,th}$ = 10 000 g/mol for DMAm and DEAm, (■) and (◆) in 50 wt. % ethanol.

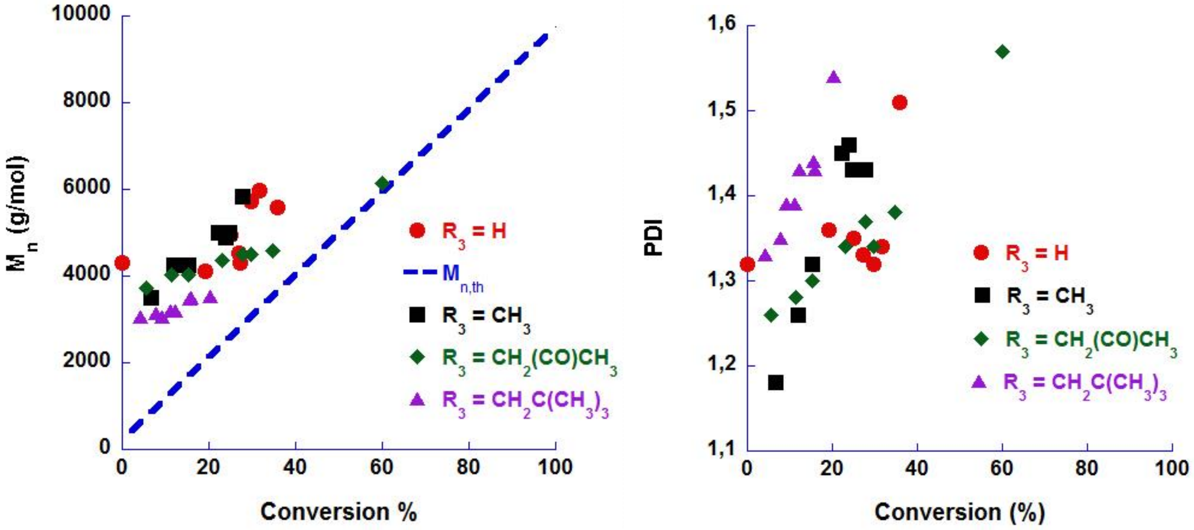

Figure S24. Evolution of M_n and PDI versus conversion during RAFT/MADIX polymerization of Type-II acrylamides in 50% wt. ethanol at 60°C.

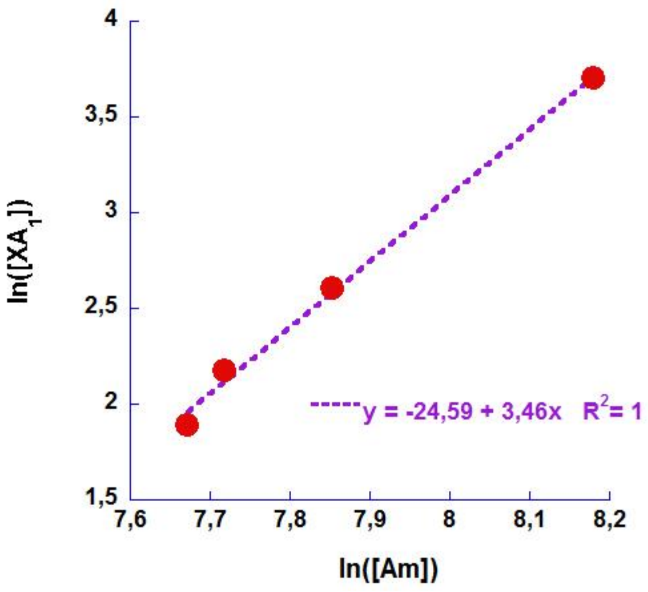

Figure S25. Double log plot of XA1 concentration vs Am concentration, $[Am]_0$ = 3.4 mol/L, $[XA1]_0$ = 1.2.10^{-2} mol/L, $[V\text{-}50]_0$ = 8.1.10^{-3} mol/L, in 25% wt. water/ethanol (4.5 : 1), T = 60°C.

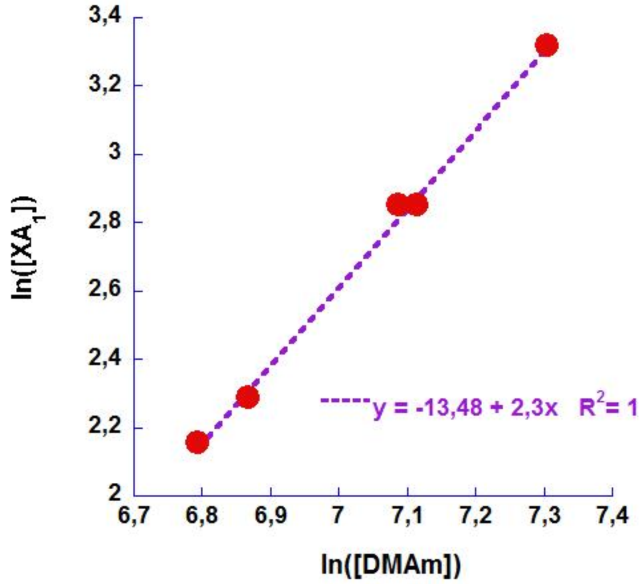

Figure S26. Double log plot of XA1 concentration vs DMAm concentration, $[DMAm]_0$ = 3.7 mol/L, $[XA1]_0$ = 9.5.10^{-2} mol/L, $[AIBN]_0$ = 8.1.10^{-3} mol/L, in 50 wt.% ethanol, T = 60°C.

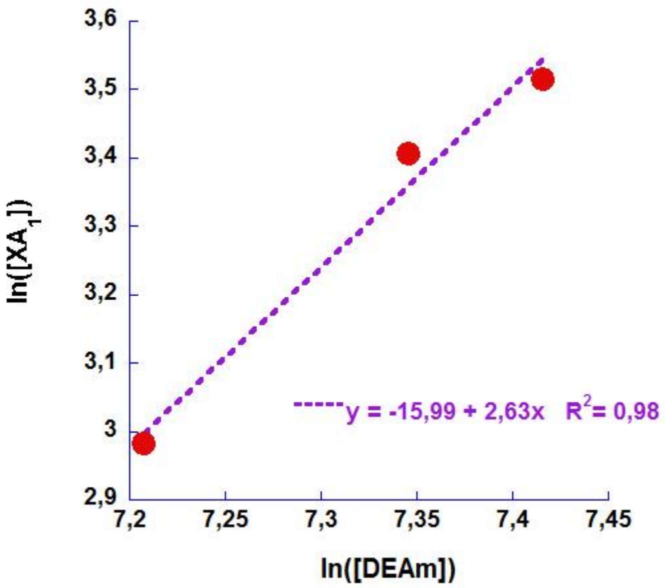

Figure S27. Double log plot of XA1 concentration vs DEAm concentration, $[DEAm]_0$ = 3.7 mol/L, $[XA_1]_0 = 9.5.10^{-2}$ mol/L, $[AIBN]_0 = 8.1.10^{-3}$ mol/L in 50 wt.% ethanol, T = 60°C.

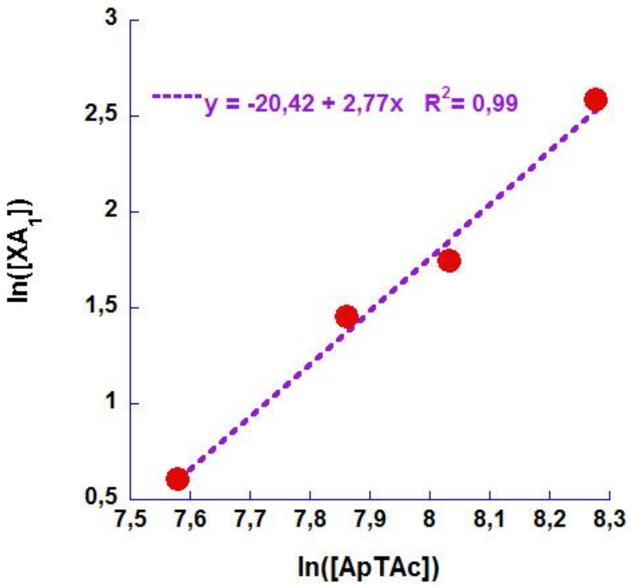

Figure S28. Double log plot of XA1 concentration vs ApTAC concentration, $[ApTAc]_0$ = 3.2 mol/L, $[XA1]_0 = 6.9.10^{-2}$ mol/L, $[V\text{-}50]_0 = 9.6.10^{-3}$ mol/L, in 50 wt.% water / ethanol (4.5 : 1), T = 60°C.

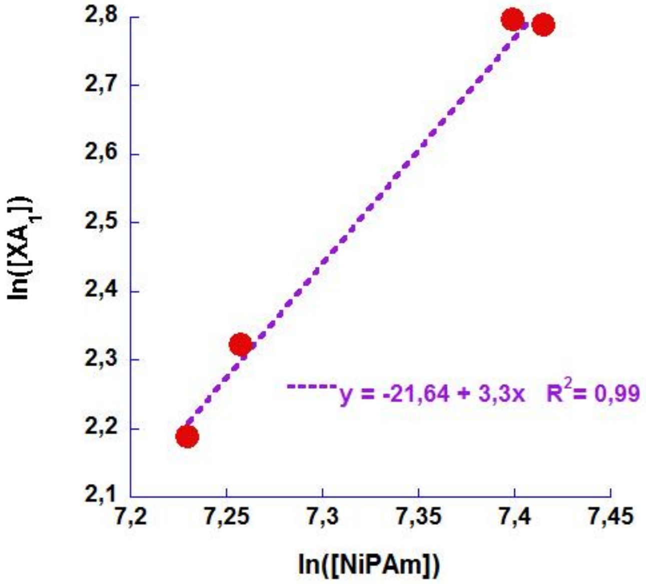

Figure S29. Double log plot of XA1 concentration vs NiPAm concentration, $[\text{NiPAm}]_0$ = 3.9 mol/L, $[\text{XA1}]_0$ = 4.5.10^{-2} mol/L, $[\text{AIBN}]_0$ = 8.5.10^{-3} mol/L, in 50 wt.% ethanol, T = 60°C.

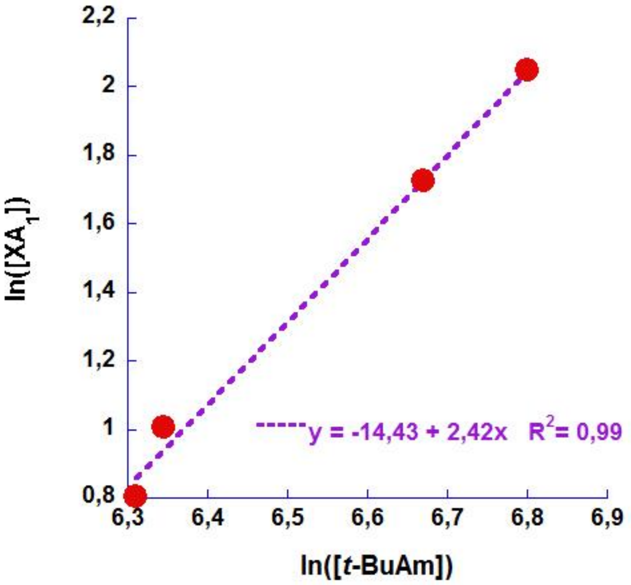

Figure S30. Double log plot of XA1 concentration vs *t*-BuAm concentration, $[t\text{-BuAm}]_0$ = 5.9 mol/L, $[\text{XA1}]_0$ = 7.7.10^{-2} mol/L, $[\text{AIBN}]_0$ = 8.5.10^{-3} mol/L, in 50 wt.% ethanol, T = 60°C.

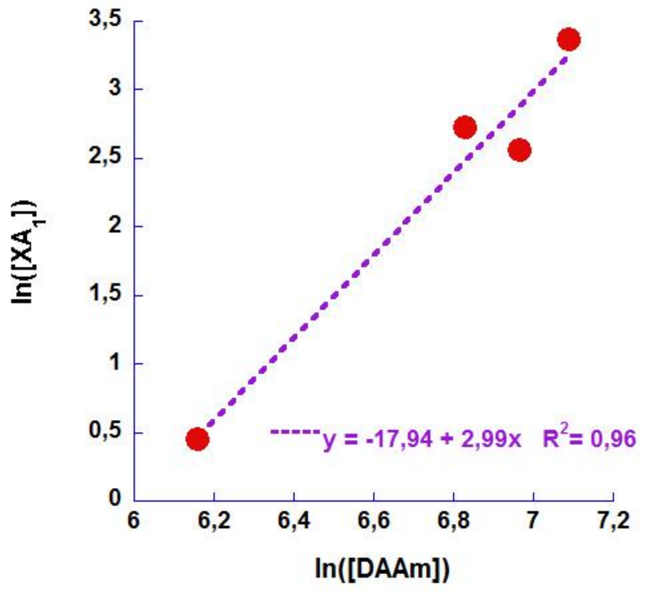

Figure S31. Double log plot of XA1 concentration vs DAAm concentration, $[DAAm]_0$ = 2.6 mol/L, $[XA1]_0 = 4.5.10^{-2}$ mol/L, $[AIBN]_0 = 4.2.10^{-3}$ mol/L, in 50 wt.% ethanol, T = 60°C.

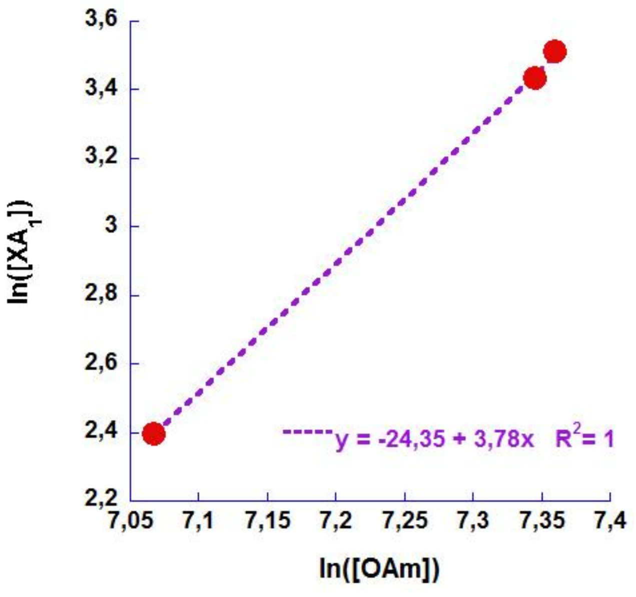

Figure S32. Double log plot of XA1 concentration vs OAm concentration, $[OAm]_0$ = 2.4 mol/L, $[XA1]_0 = 4.5.10^{-2}$ mol/L, $[AIBN]_0 = 3.8.10^{-3}$ mol/L, in 50 wt.% ethanol, T = 60°C.

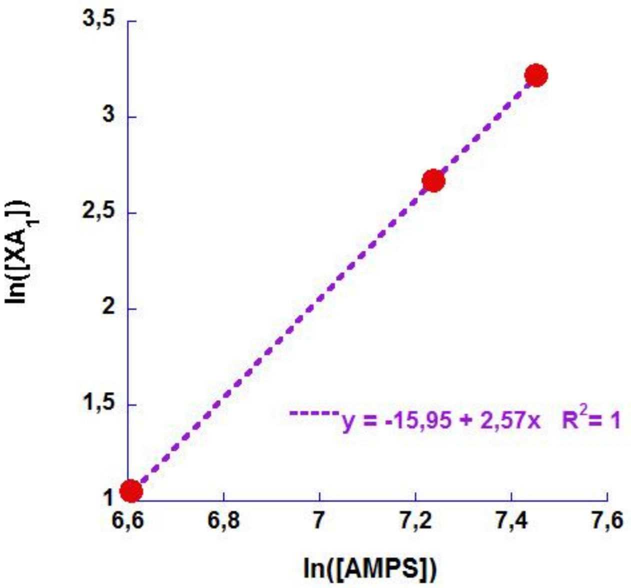

Figure S33. Double log plot of XA1 concentration vs AMPS concentration, $[AMPS]_0$ = 1.8 mol/L, $[XA1]_0$ = 4.0.10^{-2} mol/L, $[ACVA]_0$ = 7.7.10^{-3} mol/L, in 40 wt.% water/ethanol (4.5 : 1), T = 60°C.

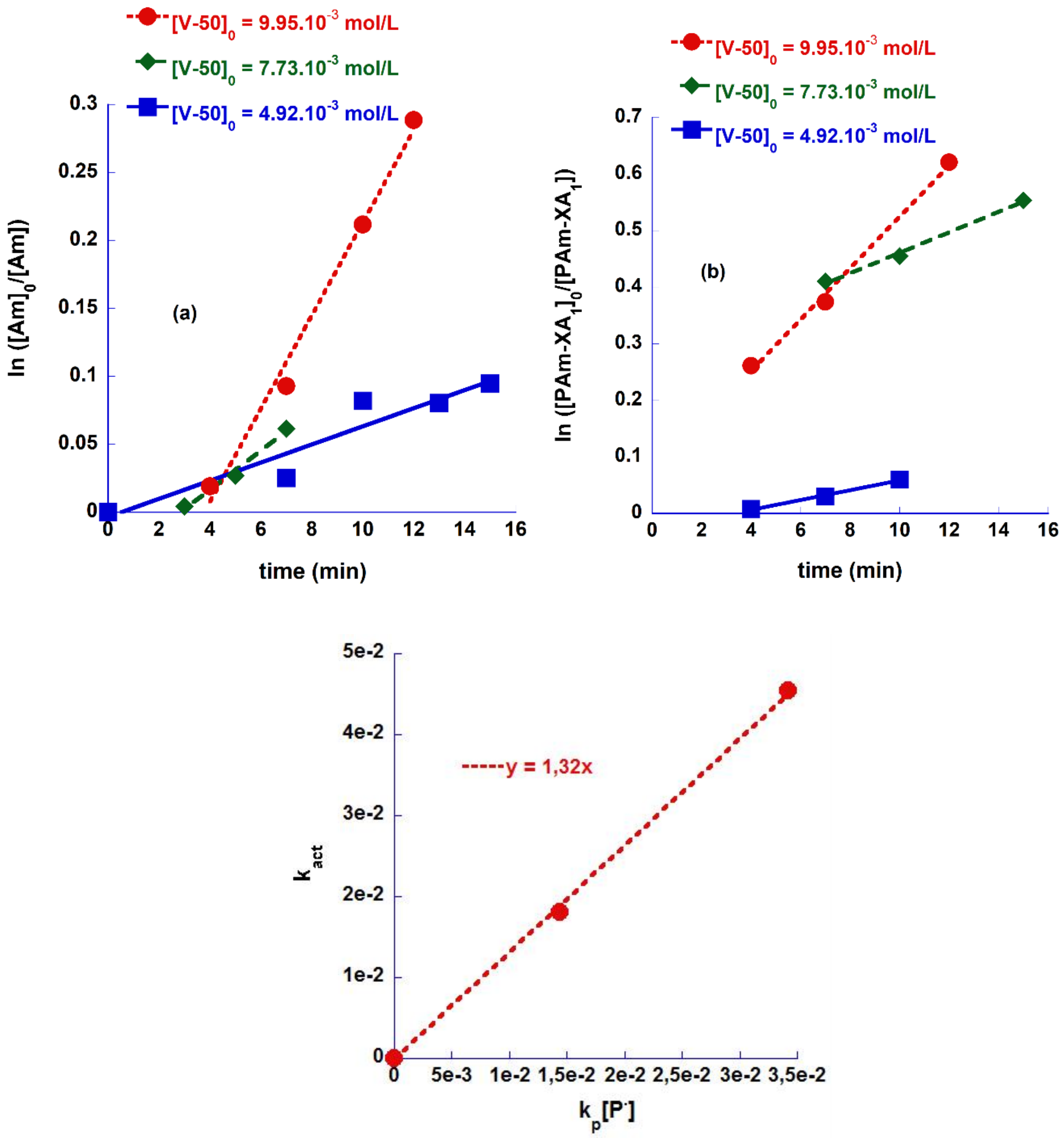

Figure S34. Determination of $C_{tr,PnXA1}$ for arylamide at 60°C (7a) Ln([M]$_0$/[M]) vs time (7b) Ln([S]$_0$/[S]) vs time (7c) Plot of k_{act} vs k_p[P·]. The leading coefficient gives $C_{tr,PnXA1}$.

References.

1. Arita, T.; Beuermann, S.; Buback, M.; Vana, P. RAFT Polymerization of Methyl Acrylate in Carbon Dioxide. *Macromol. Mater. Eng.* **2005**, *290*, 283–293, doi:10.1002/mame.200400274.
2. Hutchinson, R.A.; Paquet, D.A.; McMinn, J.H. Determination of Free-Radical Chain-Transfer Rate Coefficients by Pulsed-Laser Polymerization. *Macromolecules* **1995**, *28*, 5655–5663, doi:10.1021/ma00120a035.
3. Beuermann, S.; Paquet, D.A.; McMinn, J.H.; Hutchinson, R.A. Determination of Free-Radical Propagation Rate Coefficients of Butyl, 2-Ethylhexyl, and Dodecyl Acrylates by Pulsed-Laser Polymerization. *Macromolecules* **1996**, *29*, 4206–4215, doi:10.1021/ma960081c.
4. Penzel, E. ; Goetz, N. Solution Properties of Polyacrylic Esters. I. Light Scattering and Viscosity Measurements in Tetrahydrofuran. *Angew. Makromol. Chem.* **1990**, *178*, 191-200, doi : 10.1002/apmc.1990.051780114.
5. Back, A.J.; Schork, F.J. Mass Transfer and Radical Flux Effects in Dispersed-phase Polymerization of Isooctyl Acrylate. *J. Appl. Polym. Sci.* **2006**, *102*, 5649–5666, doi:10.1002/app.25029.
6. Molyneux, P. *Water-Soluble Synthetic Polymers: Properties and Behavior, Volume 2*; CRC Press : Tallahassee, USA, 1993.
7. Klein, J.; Conrad, K.-D. Characterization of Poly(Acrylamide) in Solution, *Makromol. Chem.* **1980**, 227-240, doi :10.002/macp.1980.021810120.
